# Remote Multicomponent Rehabilitation and Cost-Effectiveness in Survivors of Critical Illness

**DOI:** 10.1001/jamanetworkopen.2026.28380

**Published:** 2026-08-10

**Authors:** Mandana Zanganeh, Brenda O’Neill, Judy M. Bradley, Bronwen Connolly, Julie Bruce, Martin Underwood, Mariam Ratna, Ranjit Lall, Chen Ji, Jill Costley, Rachel Clarke, Paul Dark, Orla Duffy, Penelope Firshman, Nigel D. Hart, Annette Henderson, Katherine Jones, Roger Kenyon, Darren Murphy, Gavin D. Perkins, Kerry Raynes, Ella Terblanche, Daniel F. McAuley, Jason Madan

**Affiliations:** 1 Warwick Clinical Trials Unit, Warwick Medical School, University of Warwick, Coventry, United Kingdom; 2 Institute of Nursing and Health Research, School of Health Sciences, Ulster University, Belfast, United Kingdom; 3 Wellcome–Wolfson Institute for Experimental Medicine, School of Medicine, Dentistry and Biomedical Sciences, Queen’s University Belfast, Belfast, United Kingdom; 4 Department of Physiotherapy, The University of Melbourne, Melbourne, Victoria, Australia; 5 University Hospitals of Coventry and Warwickshire, Coventry, United Kingdom; 6 University Hospitals Plymouth NHS Trust, Derriford Hospital, Plymouth, United Kingdom; 7 Division of Immunology, Immunity to Infection and Respiratory Medicine, Faculty of Biology, Medicine and Health, University of Manchester, Manchester, United Kingdom; 8 Surrey and Sussex Healthcare NHS Trust, Redhill, United Kingdom; 9 Patient Advisory Group, iRehab Trial Management Group, Preston, United Kingdom; 10 School of Health Rehabilitation Sciences, Health Sciences University, Bournemouth, United Kingdom

## Abstract

**Importance:**

The cost-effectiveness of remote rehabilitation for survivors following critical illness after intensive care unit (ICU) care is unknown.

**Objective:**

To evaluate the cost-effectiveness of remote multicomponent rehabilitation compared with standard care following discharge from hospital after an ICU admission.

**Design, Setting, and Participants:**

This economic evaluation was conducted within a pragmatic, multicenter, assessor-blinded trial comparing remote rehabilitation delivered online with standard care after discharge from ICU from both National Health Service (NHS) and Personal Social Services (PSS) and societal perspectives over a 6-month time horizon. The trial was conducted from December 2022 to November 2025. The setting was 52 NHS hospitals in the United Kingdom. Participants were adults (aged ≥18 years) within 12 weeks of discharge from hospital that included an ICU admission for critical illness, requiring mechanical ventilation for 48 hours or longer.

**Interventions:**

A remotely delivered rehabilitation program or standard care.

**Main Outcomes and Measures:**

Costs including using questionnaires and microcosting approach (in 2024 UK pounds sterling) and quality-adjusted life-years (QALYs), derived directly from trial data, were calculated per group and reported in terms of incremental cost per QALY gained.

**Results:**

A total of 429 participants (245 men [57%]; mean [SD] age, 55.4 [13.9] years) were enrolled, including 231 (54%) in the intervention group and 198 (46%) in the standard group. From a UK NHS-PSS perspective, the rehabilitation intervention was associated with increased mean costs (£1250; 95% CI, £562-£1938) and QALYs (0.023; 95% CI, 0.007-0.040) per participant, compared with the standard care group. Incremental cost-effectiveness ratio (ICER) was £54 034 per QALY. The probability of rehabilitation intervention being cost-effective was 3% and 11% at UK willingness-to-pay thresholds of £20 000 and £30 000 per QALY, respectively. The intervention was cost-effective for patients with mechanical ventilation for 7 days or less (ICER, £21 476 per QALY) or if a societal perspective was adopted (ICER, £6341 per QALY).

**Conclusions and Relevance:**

In this economic analysis, among ICU survivors overall, a remotely delivered multicomponent rehabilitation program was not cost-effective from a UK NHS-PSS perspective. Cost-effectiveness was more favorable from a societal perspective and for patients receiving mechanical ventilation for 7 days or less. For rehabilitation interventions to be both clinically and cost-effective a precision medicine approach to medical and psychosocial health care interventions is needed once patients are home from hospital.

## Key Points

**Question:**

Is a remote multicomponent rehabilitation intervention cost-effective compared with standard care after critical illness?

**Findings:**

This economic evaluation, conducted from a National Health Service and Personal Social Service perspective and including 429 participants over 6 months, found that rehabilitation was associated with a statistically significant increase of 0.023 quality-adjusted life-years (QALYs) and a statistically significant increase in costs of £1250 compared with standard care. The incremental cost-effectiveness ratio was £54 034 per QALY, whereas that for patients undergoing mechanical ventilation for 7 days or less was £21 476 per QALY.

**Meaning:**

These findings suggest that remote rehabilitation is cost-effective, according to UK thresholds, only for patients undergoing mechanical ventilation for 7 days or less.

## Teaser

This economic evaluation evaluates the cost-effectiveness of remote multicomponent rehabilitation vs standard care following discharge from hospital after an intensive care unit admission in the United Kingdom.

## Introduction

The consequences of critical illness are substantial, including high costs to society and health care systems incurred after patients are discharged home from hospital. Hospital readmissions^1,2^ are common, particularly in the first 6 months after hospital discharge. Substantial use of health and social care is reported postdischarge, and many intensive care unit (ICU) survivors fail to return to full-time employment.^3,4,5^

The literature to support remote multicomponent rehabilitation for critical care survivors is limited, and the cost-effectiveness of remote rehabilitation for survivors following critical illness after ICU care is unknown. To address this gap, we conducted a preplanned economic evaluation from both National Health Service (NHS) and Personal Social Services (PSS) and societal perspectives alongside the iRehab multicenter randomized clinical trial. Our aim was to determine the costs, outcomes (quality-adjusted life-years [QALYs]) and overall cost-effectiveness of a 6-week remote multicomponent personalized rehabilitation intervention delivered online to people discharged from ICU compared with standard care.

## Methods

### Trial Design, Setting, and Participants

The iRehab study protocol has been published previously.^6^ This pragmatic, open-label, assessor blind, multicenter, randomized clinical trial recruited from UK NHS hospitals (see eAppendix 1 in Supplement 1 for sites). The trial was conducted from December 2022 to November 2025. Patient and public involvement was embedded throughout the trial with representation on the iRehab Trial Management Group. Ethical approval was granted by the London Central Research Ethics Committee. The trial was sponsored by Ulster University and coordinated by the Warwick Clinical Trials Unit. Participants were adults (aged ≥18 years) who had received invasive mechanical ventilation for 48 hours or longer and were up to 12 weeks after hospital discharge. Informed consent was obtained remotely by a member of the hospital research team once potential study participants had returned home.

### Randomization and Data Collection

Randomization, to either the rehabilitation intervention or standard care, used a minimization algorithm stratified by (1) hospital site and (2) duration of invasive mechanical ventilation (≤7 days vs >7 days). Participants completed online questionnaires at baseline, 8 weeks, and 6 months. Telephone or postal options for questionnaire were available.

### Trial Interventions

The iRehab intervention was a multicomponent intervention delivered remotely by a trained intervention specialist over 6 weeks (described in detail elsewhere).^7^ In brief, it included 4 core components: (1) weekly symptom management, (2) exercise and physical activity, (3) psychological support, and (4) group-based peer support sessions. Participants attended weekly one-to-one remote sessions and were also invited to attend weekly group-based remote exercise and peer support sessions. The comparator was standard NHS care, which typically included a single brief contact (phone call or face-to-face) with highly variable content and some signposting to other services; sites offering structured post-ICU rehabilitation were excluded.

### Economic Evaluation Methods

The economic evaluation took the form of a cost-utility analysis using incremental costs per QALY gained as a primary outcome and followed the Consolidated Health Economic Evaluation Reporting Standards (CHEERS) reporting guideline.^8^ The perspective of the base-case analysis was that of the UK NHS-PSS^9^ following intention-to-treat principles. Additional sensitivity analyses were undertaken to explore different sources of data for particular cost categories, including societal costs and alternative health-related quality of life (HRQoL) value set. The time horizon of this within-trial analysis matched the length of the iRehab follow-up. As follow-up continued only for 6 months, costs and benefits were not discounted.^9^ Analyses were performed in accordance with the aims and methods specified in the iRehab trial protocol^6^ and the health economics analysis plan.

### Cost Measures

The costs of the intervention were obtained through a microcosting analysis^10,11^ (eTable 1 in Supplement 1). Resource use and costs for both comparators were captured using resource use questionnaires. Self-reported data were collected from participants on NHS-PSS and societal use during the 6-month period following randomization (Table 1). Data regarding inpatient admissions were also collected by the sites. We conducted an analysis of the sensitivity of health economic results to the source of data (self-report or local electronic records) regarding inpatient admissions.

**Table 1.** Unit Cost of Health Care Services and Resources Used by iRehab Participants

Service	Unit cost, £^a^	Source
Rehabilitation intervention	NHS-PSS perspective, 337 per patient in the intervention group; societal perspective, 420 per patient in the intervention group	University of Warwick; Ulster University; Unit Costs of Health and Social Care 2024^10^; National Minimum Wage and National Living Wage rates 2024^11^
NHS-PSS perspective		
Inpatient		
Admitted overnight to hospital	Elective inpatient stays, 6215 average cost per episode; day cases, 1031 average cost per day; regular day or night, 402 average cost per day; nonelective inpatient stays (long stays), 5134 average cost per episode; nonelective inpatient stays (short stays), 792 average cost per episode; day adult critical care, 2276 average cost per day; accident and emergency, 273 for up to 24-hour service	National Cost Collection Data Publication: National Schedule 2023-2024^12^
Nursing home (stayed overnight)	224 average cost per day	Unit Costs of Health and Social Care 2024^10^
Outpatient		
Hospital outpatient visits	Outpatient procedures or visits, 230 per attendance	National Cost Collection Data Publication: National Schedule 2023-2024^12^
Accident and emergency visits	273 per attendance	National Cost Collection Data Publication: National Schedule 2023-2024^12^
Nursing home (day care)	87 per attendance	Unit Costs of Health and Social Care 2024^10^
Primary and community care		
Visit to GP in the medical center	222 per hour; 3.7 per minute	Unit Costs of Health and Social Care 2024^10^
GP doing a home visit	222 per hour; 3.7 per minute plus travel^b^	
GP telephone contact	9.8 per call	
GP video consultation	222 per hour; 3.7 per minute	
Practice nurse contact	58 per hour; 1 per minute	
District nurse, NHS	57 per hour; 1 per minute	
Telephone calls to NHS Direct	14.3 per call^51^	
Calls for ambulance or paramedic	250 per service^12^	
Occupational therapy contacts or visits	68 per hour; 1.1 per minute	
Physiotherapy contacts or visits	41 per hour, 0.7 per minute	
Dietetics	62 per hour, 1 per minute	
Speech and language	100 per hour one to one (1.7 per minute); 55 per hour group (0.9 per minute)	
Psychology or mental health services	69 per hour; 1.1 per minute	
Other		
Chiropodist	54 per hour; 0.9 per minute	
Podiatrist	54 per hour; 0.9 per minute	
Social services		
Meals on wheels	27 per hour	Unit Costs of Health and Social Care 2024^10^
Laundry service	27 per hour	
Social worker contacts	48 per hour	
Care worker contacts, including help at home	27 per hour	
Other (eg, care company)	27 per hour	
Special equipment or aids	Unit cost based on type	NHS Supply Chain (Aids for Daily Living) 2024^13^; Unit Costs of Health and Social Care 2024^10^
Medication	Cost per item	National Prescription Cost Analysis 2023 to 2024^14^
Societal perspective		
Private costs		
Special equipment or aids	Related costs	Different costs were stated by patients
Private Health Services	Costs related to physiotherapy, psychology, private care home, private carer, childcare, other	Different costs were stated by patients
Traveling to health service providers	Costs related to travel to health service providers (eg, car, public transport, parking fees, taxi)	Different costs were stated by patients
Work absence		
Time off work: participant	Unit cost per day	Office for National Statistics (Labour market overview, UK: Employee earnings) 2024^15^
Time off work: relatives and friends	Unit cost per hour	Office for National Statistics (Labour market overview, UK: Employee earnings) 2024^15^
Extra costs to relatives and friends	Costs related to travel to health service providers (eg, car, public transport, parking fees, taxi), childcare, help with housework, other	Different costs were stated by patients

Abbreviations: GP, general practitioner; NHS, National Health Service; PSS, Personal Social Service.

^a^ Costs are reported in 2024 UK pounds sterling. See eTables 1 to 8 in Supplement 1 for more details on how costs were calculated.

^b^ Twelve minutes travel time (£44.4) and 5 miles travel cost (NHS travel reimbursed at 56 p per mile) (£2.80) = £47.2.

Individual participant’s costs were estimated in UK pounds sterling multiplying resources use by their reference costs, reflated to 2024 values. Costs of inpatient admissions, outpatient visits, and accident and emergency visits were estimated using the National Schedule of Reference Costs 2023 to 2024^12^ (eTables 2-4 in Supplement 1). Nursing home, primary and community care, and social services were costed using the Unit Costs of Health and Social Care 2024.^10^ Equipment and aids used were costed using the NHS Supply Chain 2024 and the Unit Costs of Health and Social Care 2024^10,13^ (eTable 5 in Supplement 1). Medications prescribed postdischarge were costed using National Prescription Cost Analysis 2023 to 2024^14^ (eTable 6 in Supplement 1). Private costs and the extra costs to relatives or friends were patient reported. Lost earnings for both participants and relatives or friends were estimated using the Office for National Statistics 2024^15^ (eTables 7 and 8 in Supplement 1). Deceased patients were assigned a cost of zero from the date of death.

### Measure of Estimated Effectiveness

The primary measure of estimated effectiveness of the iRehab economic evaluation was the QALY. QALYs are a widely used and recommended^9^ metric that combine quantity (length) of life and preference-based HRQoL into a single value. HRQoL was obtained through participants’ responses to the EuroQol EQ-5 dimension-5 level (EQ-5D-5L) instrument.^16^ For the base-case analysis, EQ-5D-5L scores were converted to health status scores using the algorithm of Hernández Alava et al^17^ recommended by National Institute for Health and Care Excellence guidance,^18^ providing a single, preference-based (utility) index. QALYs were calculated as the area under the curve connecting utility scores reported at different time points.^19^ Deceased patients were assigned a utility of zero from the date of death. As a sensitivity analysis, we used the value set of Devlin et al^20^ to calculate utilities and QALYs.

### Statistical Analysis

The intention-to-treat principle was adopted.^21^ The unadjusted calculated mean per-patient values were given for the intervention and control groups alongside mean difference (MD) and 95% CIs obtained through nonparametric methods using 2000 replications.^22^ The distributions of the calculated costs and QALYs were interrogated using graphs and skewness statistics, and generalized linear regression models were used.^23^

The missing utility and different cost components data were imputed under the missing at random assumption, at each time point, using fully conditional multiple imputation by chained equations^24^ (eAppendix 2 in Supplement 1). The multiple imputation model used covariates^6^ and utility scores collected at baseline. Fifty imputations were conducted separately by trial group.^25^ Imputed dataset was used for the base case analysis to determine the incremental costs and incremental QALYs. Adjustment for baseline utility and for the same covariates as for the primary outcome was made.^26,27^ Cost-utility is presented as an incremental cost-effectiveness ratio (ICER), calculated as difference in costs between comparators over difference in QALYs between comparators.^28^ Measures of uncertainty were calculated for the MD estimates through bootstrap methods using 2000 replications.^22^ Simulated cost-QALY pairs were depicted on a cost-effectiveness plane and were plotted as cost-effectiveness acceptability curves.^29^ Net monetary benefits (NMBs) were estimated for a range of different UK willingness-to-pay (WTP) thresholds. The expected value of perfect information was computed from the NMB data and presented graphically. Data management tasks and statistical analyses were performed in Stata statistical software version 19.5 (StataCorp). Statistical significance was assessed using a 2-sided *t* test, with a significance level of *P* < .05. The base-case analysis was complemented by a series of additional sensitivity analyses and subgroup analyses (eAppendix 3 in Supplement 1).

## Results

### Participant Characteristics

Between November 18, 2022, and April 14, 2025, we screened 3705 potential participants. Of these, 429 patients were enrolled from 52 sites (231 [54%] assigned to the intervention group and 198 [46%] to the standard group), for a maximum follow-up period of 6 months. There were 245 men (57%; mean [SD] age, 55.4 [13.9] years), with a mean (SD) Acute Physiology and Chronic Health Evaluation II score of 18.4 (8.1), and mean (SD) length of stay in the ICU of 18.8 (15.8) days. Participants in both groups had similar baseline characteristics. The study recruitment process is summarized elsewhere.^6^

### Data Completeness

The availability of key economic data by comparator is provided in eTable 9 in Supplement 1. Missingness for both cost (all resource use categories) and QALY (all EQ-5D-5L measurements) exceeded 5% over the 6 months, which made imputation of missing data necessary.

### Cost Analysis

Costs for each resource use category and assessed comparator for the complete cases (unadjusted) are given in Table 2. The findings show that, from the NHS-PSS perspective, the rehabilitation intervention was not associated with significantly greater costs for the cost categories of inpatient admissions (MD, £977.67; 95% CI, £−77.02 to £2032.36; 35 vs 47 admissions in standard care vs intervention groups), primary and community care (MD, £105.76; 95% CI, £−51.50 to £263.02), outpatient visits (MD, £68.26; 95% CI, £−98.37 to £234.88), accident and emergency visits (MD, £12.11; 95% CI, £−54.98 to £79.20), special equipment and aids (MD, £7.38; 95% CI, £−36.95 to £51.73), and outpatient nursing home visits (MD, £7.13; 95% CI, £−3.93 to £18.19) compared with the standard care group. Conversely, rehabilitation intervention was not associated with significant cost savings for the cost categories of medications (MD, −£15.25; 95% CI, £−65.19 to £34.68) and social services (MD, −£7.64; 95% CI, £−49.68 to £34.40) compared with the standard care group. All findings of societal costs are more favorable to the intervention group; the largest cost saving was for participants time off work, but the MD was not significant (MD, −£1134.87; 95% CI, £−2789.05 to £519.30). Overall, from the NHS-PSS perspective over 6 months including intervention cost of £337, the mean per-patient cost with rehabilitation intervention was not significantly higher (MD, £912.49; 95% CI, −£39.06 to £1864.04; *P* = .06) compared with the standard care group. From the societal perspective over 6 months including intervention cost of £420, the mean per-patient cost with rehabilitation intervention was not significantly lower (MD, −£904.74; 95% CI, −£3500.16 to £1690.68; *P* = .49) compared with the standard care group. Using site-reported inpatient admissions data did not change the overall cost materially from either NHS-PSS and societal perspectives (eTable 10 in Supplement 1). Detailed information regarding patient-reported and site-reported inpatient admissions is provided in eTables 11 and 12 in Supplement 1.

**Table 2.** NHS-PSS and Societal Costs for Resources by Patient-Reported Health Status^a^

Variable	Standard care		Rehabilitation intervention		Mean difference (95% CI)^b^	*P* value^b^
	No. of patients who provided data	Mean (SD)	No. of patients who provided data	Mean (SD)		
Health status, QALYs^c^						
Baseline utilities	198	0.532 (0.268)	231	0.501 (0.315)	−0.031 (−0.085 to 0.023)	.27
8-wk Utilities	167	0.582 (0.312)	178	0.600 (0.297)	0.018 (−0.045 to 0.081)	.58
6-mo Utilities	148	0.596 (0.332)	169	0.626 (0.305)	0.030 (−0.041 to 0.101)	.41
Area under the curve	143	0.298 (0.139)	161	0.300 (0.135)	0.002 (−0.028 to 0.033)	.87
Costs for resource use categories, £^d^						
NHS-PSS						
Inpatient						
Inpatient admissions^e^	129	900.95 (3173.17)	127	1878.62 (5261.71)	977.67 (−77.02 to 2032.36)	.07
Inpatient nursing home admissions	127	0	122	0	0	NA
Outpatient						
Outpatient visits	126	766.30 (626.39)	125	834.56 (739.71)	68.26 (−98.37 to 234.88)	.42
Accident and emergency visits	128	102.37 (229.82)	124	114.48 (307.64)	12.11 (−54.98 to 79.20)	.72
Outpatient nursing home visits	127	0 (0)	122	7.13 (78.77)	7.13 (−3.93 to 18.19)	.21
Primary and community care	125	404.74 (546.84)	124	510.50 (718.83)	105.76 (−51.50 to 263.02)	.19
Social services	122	31.06 (171.70)	123	23.41 (161.89)	−7.64 (−49.68 to 34.40)	.72
Special equipment and aids	122	85.45 (168.31)	122	92.84 (179.94)	7.38 (−36.95 to 51.73)	.74
Medications	121	94.95 (245.45)	120	79.69 (146.03)	−15.25 (−65.19 to 34.68)	.55
Societal						
Private costs						
Special equipment and aids	122	110.13 (617.08)	122	81.83 (361.65)	−28.30 (−155.03 to 98.43)	.66
Private Health Services	121	53.64 (197.15)	122	40.23 (205.11)	−13.41 (−63.03 to 36.21)	.60
Traveling to health service practitioners	119	71.13 (288.75)	119	45.74 (123.00)	−25.39 (−81.46 to 30.68)	.38
Work absence						
Participant taken time off from work	121	4601.31 (7325.69)	123	3466.43 (6045.01)	−1134.87 (−2789.05 to 519.30)	.18
Relatives or friends taken time off from work	122	1375.31 (4245.45)	119	1053.60 (2788.37)	−321.71 (−1221.79 to 578.38)	.48
Extra costs to relatives or friends	121	167.63 (777.53)	121	69.56 (420.19)	−98.07 (−250.55 to 54.42)	.21
All resource use costs (NHS-PSS perspective)						
Randomization to 8 wk	140	1282.48 (2852.58)	144	1562.32 (2716.96)	279.84 (−369.68 to 929.37)	.40
8 wk to 6 mo	127	1104.26 (1741.18)	132	1537.94 (2704.98)	433.68 (−119.08 to 986.44)	.12
Total follow-up period	118	2434.92 (3686.14)	115	3010.41 (3667.22)	575.49 (−355.85 to 1506.83)	.23
Total follow-up period plus intervention cost (£337)	118	2434.92 (3686.14)	115	3347.41 (3667.22)	912.49 (−39.06 to 1864.04)	.06
All resource use costs (societal)						
Randomization to 8 wk	136	3148.57 (3875.93)	144	2746.88 (3552.37)	−401.69 (−1280.64 to 477.25)	.37
8 wk to 6 mo	129	3217.49 (6647.99)	132	2379.19 (4771.97)	−838.29 (−2260.22 to 583.63)	.25
Total follow-up period	116	6638.34 (9759.08)	115	4851.60 (7362.55)	−1786.74 (−4067.52 to 494.04)	.13
Total follow-up period plus intervention cost (£83)	116	6638.34 (9759.08)	115	4934.60 (7362.5)	−1703.74 (−3885.62 to 478.15)	.13
All resource use costs (societal perspective)						
Randomization to 8 wk	134	4500.41 (5244.48)	140	4199.39 (4068.88)	−301.02 (−1416.70 to 814.65)	.60
8 wk to 6 mo	126	4342.80 (7086.22)	129	3906.20 (6158.75)	−436.60 (−2093.86 to 1220.65)	.61
Total follow-up period	113	9215.84 (10 847.5)	112	7891.10 (814.67)	−1324.74 (−3870.54 to 1221.06)	.31
Total follow-up period plus intervention cost (£420)	113	9215.84 (10 847.5)	112	8311.10 (8814.67)	−£904.74 (−3500.16 to 1690.68)	.49

Abbreviations: NA, not applicable; NHS-PSS, National Health Service and Personal Social Service; QALYs, quality-adjusted life-years.

^a^ Analysis includes 3 patients (1 from rehabilitation and 2 from standard care) who died by the 6-month follow-up time point.

^b^ Mean difference (95% CI) and *P* value were calculated from bootstrapping using 2000 replications.

^c^ Health status (determined with the EuroQol EQ 5-Dimension 5-Level instrument) was calculated using the value set of Hernández Alava et al.^17^

^d^ Costs are reported in 2024 UK pounds sterling.

^e^ Includes patient-reported admissions. There were 35 admissions in the standard care group and 47 admissions in the rehabilitation intervention group. Inpatient admissions for rehabilitation intervention group includes 2 serious adverse events.

### Estimated Effectiveness

Table 2 summarizes the health outcomes and between-group MDs at each time point and across the follow-up period for the complete cases (unadjusted). EQ-5D utility scores from follow-up time point 2 suggest a small but nonsignificant gain for the rehabilitation intervention group. Overall, using the algorithm of Hernández Alava et al,^17^ over 6 months, there was a small, nonsignificant QALY gain for the rehabilitation intervention (MD, 0.002; 95% CI, −0.028 to 0.033; *P* = .87) compared with the standard care group. Using the value set of Devlin et al,^20^ over 6 months, the QALY results were similar with a small, nonsignificant QALY gain for the rehabilitation intervention (MD, 0.003; 95% CI, −0.024 to 0.031; *P* = .81) compared with the standard care group (eTable 10 in Supplement 1). The findings of the complete cases (adjusted) are provided in eTable 13 in Supplement 1.

### Base-Case Cost-Utility Analysis

Results for the base-case analysis (UK NHS-PSS perspective) are presented in Table 3. Over 6 months, rehabilitation intervention was associated with a higher total per-patient cost (adjusted MD, £1250; 95% CI, £562-£1938) compared with the standard care group. In terms of QALYs gained, rehabilitation intervention appeared to be modestly more effective than standard care, resulting in a gain of 0.023 QALYs (95% CI, 0.007-0.040). On average, rehabilitation intervention was more costly and modestly more effective than standard care (ICER of £54 034 per QALY).

**Table 3.** Cost-Utility Analysis: Base-Case, Sensitivity, and Subgroup Analyses Results at 6 Months^a^

Analysis	Incremental cost, mean (95% CI), £	Incremental QALYs (95% CI)	ICER, £ per QALY	Probability of cost-effectiveness		NMB, £		EVPI, £	
				WTP £20 000 per QALY	WTP £30 000 per QALY	WTP £20 000 per QALY	WTP £30 000 per QALY	WTP £20 000 per QALY	WTP £30 000 per QALY
Base-case analysis: NHS-PSS perspective, imputed costs and QALYs with adjustment for baseline utility and covariates	1250 (562 to 1938)	0.023 (0.007 to 0.040)	54 034	0.03	0.11	−787	−556	5	26
Sensitivity analyses									
Societal perspective, imputed costs and QALYs with adjustment for baseline utility and covariates	147 (−1482 to 1775)	0.023 (0.007 to 0.040)	6341	0.64	0.72	316	547	215	153
Imputed costs and QALYs with adjustment for baseline utility and covariates, using site-reported inpatient admissions costs	1270 (623 to 1918)	0.023 (0.007 to 0.040)	54 919	0.02	0.09	−808	−576	2	17
Imputed costs and QALYs with adjustment for baseline utility and covariates, using Devlin et al^20^ algorithm	1250 (562 to 1938)	0.023 (0.008 to 0.038)	54 023	0.03	0.10	−787	−556	4	24
Imputed costs and QALYs with adjustment for baseline utility and covariates, excluding intervention costs	913 (225 to 1601)	0.023 (0.007 to 0.040)	39 463	0.13	0.31	−450	−219	30	95
Imputed costs and QALYs with adjustment for baseline utility	1419 (635 to 2203)	0.021 (0.005 to 0.037)	68 208	0.01	0.05	−1003	−795	1	9
Complete cases costs and QALYs with adjustment for baseline utility and covariates	958 (−16 to 1933)	0.022 (0.001 to 0.041)	45 975	0.14	0.27	−541	−333	44	99
Subgroup analysis									
Imputed costs and QALYs with adjustment for baseline utility and covariates, include only patients with ventilation duration ≤7 d	980 (−204 to 2164)	0.046 (0.017 to 0.074)	21 476	0.47	0.67	−67	389	261	174
Imputed costs and QALYs with adjustment for baseline utility and covariates, include only patients with ventilation duration >7 d	1783 (815 to 2751)	0.013 (−0.013 to 0.038)	142 794	0.00	0.01	−1533	−1409	1	3

Abbreviations: EVPI, expected value of perfect information; ICER, incremental cost-effectiveness ratio; NHS-PSS, National Health Service and Personal Social Service; NMB, net monetary benefits; QALYs, quality-adjusted life-years; WTP, willingness to pay.

^a^ Estimates were derived from bootstrapping using 2000 replications (rehabilitation intervention vs standard care). Analyses were based on a generalized linear regression model adjusted for baseline utility score, stratification variables (ie, hospital sites and ventilation duration), and other covariates (ie, age, gender, ethnicity, and mobility). Costs are given in 2024 UK pounds sterling.

**Figure 1.**
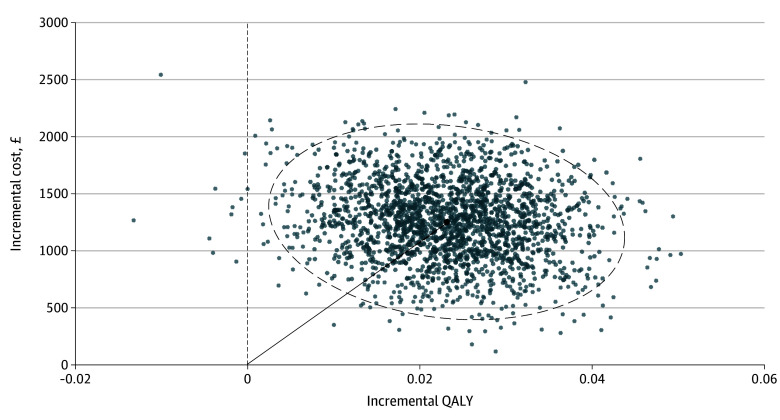
Cost-Effectiveness Plane Depicting the Distribution of Simulated Cost and Quality-Adjusted Life-Years (QALY) Pairs From the National Health Service, Personal Social Service Perspective Costs are given in 2024 UK pounds sterling.

**Figure 2.**
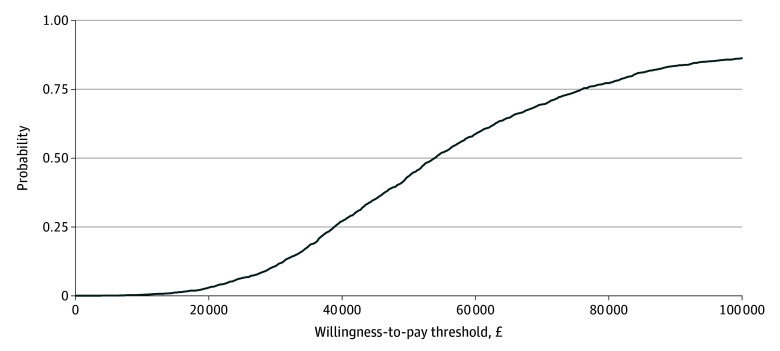
Cost-Effectiveness Acceptability Curve Showing the Probability of Rehabilitation Intervention Being Cost-Effective at Different Values of Willingness-to-Pay for a Quality-Adjusted Life-Year From the National Health Service, Personal Social Service Perspective

Figure 1 shows that approximately 99.5% of the simulated pairs are located in the northeast quadrant of the plane, indicating that rehabilitation intervention is more costly and more effective than standard care. Figure 2 shows that at £20 000 per QALY, the probability that rehabilitation intervention is cost-effective is 3%; at £30 000 per QALY, it increases to 11%. At the WTP threshold of £20 000 per additional QALY, the incremental NMB is −£787, and it increases to −£556 at the WTP threshold of £30 000 per additional QALY. A negative incremental NMB indicates that, at a particular WTP threshold, rehabilitation intervention is not cost-effective (eFigure 1 in Supplement 1). The expected value of perfect information metric quantifies the monetary value of eliminating all uncertainty from the cost-effectiveness estimate across a range of WTP thresholds (eFigure 2 in Supplement 1). Overall, the base-case findings demonstrate that the rehabilitation intervention is not cost-effective.

### Sensitivity and Subgroup Cost-Utility Analyses

Apart from the complete-case analysis, all results are based on imputed and adjusted data (Table 3). From a societal perspective, rehabilitation intervention was found to be slightly more costly and modestly more effective than standard care (ICER, £6341 per QALY) (eFigure 3 in Supplement 1). A preplanned subgroup analysis found that, among participants with mechanical ventilation for 7 days or less, the ICER was £21 476 per QALY (eFigure 4 in Supplement 1). For those undergoing ventilation for more than 7 days, the ICER was £142 794 per QALY (eFigure 5 in Supplement 1). Compared with the missing at random base-case analysis, cost-effectiveness conclusions were unchanged across both missing-not-at-random scenarios, indicating robustness to plausible violations of the missing at random assumption; full results are reported in eTable 14 in Supplement 1. The overall conclusion was insensitive to all other sensitivity analyses.

## Discussion

This economic evaluation study evaluated the cost-effectiveness of remote multicomponent rehabilitation for survivors of critical illness after hospital discharge, using patient-level data collected within a pragmatic randomized clinical trial. Overall, the intervention was not cost-effective from a UK NHS-PSS perspective. Although the adjusted incremental QALY gain reached statistical significance (0.023 QALYs), it was modest, which informs our findings in terms of lack of cost-effectiveness. However, this improvement may be important for this population with high morbidity after hospital discharge.^30^ To achieve an ICER of £20 000 per QALY, the incremental cost would need to fall by £790 (63% of the total incremental cost of £1250). Given that hospitalization was the largest cost driver in the complete-case analysis (MD, £977), a substantial reduction in hospitalization costs would likely be required.

Cost-effectiveness was more favorable from a societal perspective. In settings with higher WTP thresholds, the intervention may be cost-effective, although other relevant factors may need to be considered. Additionally, the intervention could be delivered by a more diverse workforce using the intervention training materials and similar to that used in a growing skilled workforce for delivery of pulmonary rehabilitation.^31^

The intervention was cost-effective for patients receiving mechanical ventilation for 7 days or less. This finding is consistent with the clinical analysis, which demonstrated a statistically significant interaction between treatment allocation and duration of ventilation for the primary outcome. The subgroup analysis is interesting as those undergoing ventilation for 7 days or less are likely to represent a population who have less morbidity after a critical illness, where a remote intervention might be more likely to be effective. Targeted delivery of the intervention to this subgroup would be both clinically effective and cost-effective. In contrast those undergoing ventilation for more than 7 days represent a population with more severe critical illness that is less modifiable and/or for whom pathophysiological changes have been proposed to impact recovery and rehabilitation.^32,33,34^ This group may need modifications to the intervention or an alternative intervention for effectiveness and cost-effectiveness.

The finding that the intervention increased both HRQoL and hospital admissions might seem counterintuitive. Hospital admissions were self-reported, and the intervention group may have gained confidence in seeking help when unwell or may have had better recall of admissions owing to higher attention from the intervention specialist who asked participants about their health weekly during the intervention. Similarly, a trial on remote monitoring found increased hospital readmission rates compared with usual care in patients aged 65 years and older, potentially because of clinical response teams sending patients back to hospital when alerted about a change in patient symptoms.^35^ The intervention might have successfully encouraged greater activity, which improved quality of life (QoL) but also increased risk of acute hospital admissions owing to exacerbation of cardiorespiratory conditions or falls. Collection of EQ-5D-5L data directly before and after readmissions could be insightful in future trials.

Post-ICU rehabilitation operates within the biopsychosocial model of health care working to improve well-being, function, ability, and QoL but largely operates without elements such as medicines management, direct medical input, and diagnostic investigation (associated with a biomedical model).^36,37,38^ Therefore, there may be less opportunity to impact hospital admissions associated with changes in disease state. Some disease-specific rehabilitation interventions, such as pulmonary rehabilitation^39,40^ and cardiac rehabilitation,^41,42^ have been shown to be cost-effective. For these, patients usually receive optimal medical management (biomedical approach) prior to commencing the rehabilitation component (biopsychosocial approach). If the ultimate goal of rehabilitation delivered after ICU discharge includes a reduction in hospital admissions, as well as improved QoL, then implementation of tailored rehabilitation programs like iRehab could be more cost-effective by embedding a precision medicine approach to medical and psychosocial health care interventions once patients are home from the hospital.^36^

Existing trials of rehabilitation delivered during ICU^43,44,45^ and on hospital wards after ICU discharge^46^ found no evidence that rehabilitation was cost-effective compared with usual care. Bakhru et al^47^ investigated a post-ICU remote intervention compared with usual care delivered to patients after hospital discharge. The study did not find significant improvements in cost, QoL, or mortality compared with usual care; also, self-reported emergency department visits were more common in the intervention group at 6 months. The authors report limitations to the trial, including fewer remote visits by video than intended and limitations with their inclusion and exclusion criteria, and they call for consideration of the complexity of the health care models in which recovery interventions are delivered.

The use of societal-level health economics data like in the iRehab trial is proposed to increase the likelihood of detecting effects and avoid missing costs and benefits.^36^ The implementation of the iRehab trial results into practice needs to consider the totality of trial outcomes, including cost-effectiveness, effectiveness, patient acceptability, and implement ability.^44,48,49^

### Strengths and Limitations

The iRehab economic evaluation was conducted from both UK NHS-PSS and societal perspectives,^36^ providing detailed new evidence on a range of relevant cost components. A broad range of number of sensitivity and subgroup analyses were performed, giving reassurance that key assumptions did not influence results. Methods used throughout the economic analysis, including the base-case and additional analyses, were in line with good practice recommendations and requirements for the allocation of health care resources in the UK.^9,28,50^

Despite these strengths, this study has certain limitations. First, this study only had a 6-month follow-up period. A longer follow-up might change our conclusions, by illustrating whether QoL gains and/or increased hospitalizations persist. On the basis of our expected value of perfect information analysis, implementation in the overall population might not be worthwhile from our primary analysis, but it might be worthwhile if a societal perspective was taken or to consider implementing intervention only in the subgroup with shorter ventilation. Second, the level of missing data was high for some resource use categories collected via resource use questionnaires. Third, the difference between the incremental QALYs observed in the trial and the estimated differences between groups in the cost-utility are notable. It is worth noting that the imbalance at baseline EQ-5D-5L utility score might have influenced the MD for QALYs when analyzing the complete cases (unadjusted). However, we imputed the data and adjusted QALYs for baseline utility and covariates. Fourth, follow-up health care use was based on patient reports and may be subject to recall bias. Fifth, the results of this analysis may not be generalizable to other populations substantially different from trial participants.

## Conclusions

In this economic analysis, among ICU survivors overall, a remotely delivered multicomponent rehabilitation program was not cost-effective from a UK NHS-PSS perspective. Cost-effectiveness was more favorable from a societal perspective and for patients receiving mechanical ventilation for 7 days or less. For rehabilitation interventions to be both clinically effective and cost-effective, a precision medicine approach to medical and psychosocial health care interventions is likely to be needed once patients are home from hospital.

## Notes

Supplement 1. eAppendix 1. iRehab sites and acknowledged committees contributing to the iRehab study eTable 1. Rehabilitation intervention costs per 231 patients eTable 2. Unit cost of hospital inpatient admissions (patient and site reported) eTable 3. Unit cost of hospital outpatient visits eTable 4. Unit cost of Accident and Emergency (A&E) visits eTable 5. Unit cost of special equipment/aids used eTable 6. Unit cost of medication used (National Prescription Cost Analysis – 2023/24) eTable 7. Unit cost of participant taken time off from work (Office for National Statistics [Labour market overview, UK: Employee earnings]) 2024 eTable 8. Unit cost of relatives/friends taken time off (Office for National Statistics [Labour market overview, UK: Employee earnings]) 2024 eAppendix 2. Justification regarding using missing at random (MAR) method for multiple imputation eAppendix 3. A series of additional sensitivity analyses and sub-group analyses eTable 9. Completeness of resource use and EQ-5D data by follow up visit eTable 10. Health status; NHS PSS and societal costs for resource (Complete cases, unadjusted: baseline to 6 months) (Site-reported inpatient admission costs and Devlin et al algorithm for EQ-5D), (£, 2024) eTable 11. Patient-reported inpatient admissions (complete cases, unadjusted) eTable 12. Site-reported inpatient admissions (complete cases, unadjusted) eTable 13. Health status (complete cases, adjusted) eTable 14. Cost utility scenario analysis of different MNAR assumptions for missing cost and effectiveness quality-of-life data: Results at 6 months (£, 2024) eFigure 1. Incremental net monetary benefit (NMB) across different WTP values for a QALY (NHS PSS perspective). eFigure 2. Expected value of perfect information across different WTP values for a QALY (NHS PSS perspective). eFigure 3. Cost-effectiveness acceptability curve showing the probability of rehabilitation intervention being cost-effective at different values of WTP for a QALY (Societal perspective). eFigure 4. Cost-effectiveness acceptability curve showing the probability of rehabilitation intervention being cost-effective at different values of WTP for a QALY (ventilation duration <=7 days). eFigure 5. Cost-effectiveness acceptability curve showing the probability of rehabilitation intervention being cost-effective at different values of WTP for a QALY (ventilation duration >7 days). eReferences

Supplement 2. iRehab Trial Investigators

Supplement 3. Data Sharing Statement

## References

[1] Kang J, Lee KM. Three-year mortality, readmission, and medical expenses in critical care survivors: a population-based cohort study. Aust Crit Care. 2024;37(2):251-257. doi:10.1016/j.aucc.2023.07.036 37574386

[2] Lone NI, Lee R, Salisbury L, . Predicting risk of unplanned hospital readmission in survivors of critical illness: a population-level cohort study. Thorax. 2019;74(11):1046-1054. doi:10.1136/thoraxjnl-2017-210822 29622692

[3] Kamdar BB, Huang M, Dinglas VD, ; National Heart, Lung, and Blood Institute Acute Respiratory Distress Syndrome Network. Joblessness and lost earnings after acute respiratory distress syndrome in a 1-year national multicenter study. Am J Respir Crit Care Med. 2017;196(8):1012-1020. doi:10.1164/rccm.201611-2327OC 28448162 PMC5649982

[4] Griffiths J, Hatch RA, Bishop J, . An exploration of social and economic outcome and associated health-related quality of life after critical illness in general intensive care unit survivors: a 12-month follow-up study. Crit Care Med. 2013;17(3):R100. doi:10.1186/cc12745 23714692 PMC3706775

[5] Su H, Fuentes AL, Chen H, . The financial impact of post intensive care syndrome. Crit Care Clin. 2025;41(1):103-119. doi:10.1016/j.ccc.2024.08.003 39547719 PMC11681988

[6] O’Neill B, Bradley JM, Connolly B, . Remote multicomponent rehabilitation compared to standard care for survivors of critical illness after hospital discharge (iRehab): a protocol for a randomised controlled assessor-blind clinical and cost-effectiveness trial. NIHR Open Res. 2025;5:29. doi:10.3310/nihropenres.13910.2 40443419 PMC12120417

[7] O’Neill B, McAuley D, Clarke R, . Development of a patient centred, structured, individually tailored, multi-component intervention to promote rehabilitation and recovery after critical illness: content, theory, and construction. NIHR Open Res. 2025;5:64. doi:10.3310/nihropenres.14023.1 40979264 PMC12444276

[8] Husereau D, Drummond M, Augustovski F, . Consolidated health economic evaluation reporting standards (CHEERS) 2022 explanation and elaboration: a report of the ISPOR CHEERS II good practices task force. Value Health. 2022;25(1):10-31. doi:10.1016/j.jval.2021.10.008 35031088

[9] National Institute for Health and Care Excellence. Guide to the methods of technology appraisal 2013. NICE. April 4, 2013. Accessed June 30, 2026. https://www.nice.org.uk/process/pmg9/chapter/foreword 27905712

[10] Jones KC, Weatherly H, Birch S, . Unit costs of health and social care 2024 manual. University of Kent. Accessed June 30, 2026. https://kar.kent.ac.uk/109563/

[11] UK Government. National minimum wage and national living wage rates 2024. Accessed June 30, 2026. https://www.gov.uk/national-minimum-wage-rates

[12] National Health Service. National cost collection data publication: national schedule 2023-2024. Accessed June 30, 2026. https://app.powerbi.com/view?r=eyJrIjoiNjI5YjA1OWMtOGNmOC00NGQzLWIxZDYtZDAyMDQ5YWVkZjU0IiwidCI6IjM3YzM1NGIyLTg1YjAtNDdmNS1iMjIyLTA3YjQ4ZDc3NGVlMyJ9

[13] National Health Service. NHS supply chain: aids for daily living. 2024. Accessed June 30, 2026. https://www.supplychain.nhs.uk/product-information/contract-launch-brief/walking-aids-and-simple-aids-for-daily-living/#delisted

[14] NHS Business Services Authority. National prescription cost analysis—England 2023/24. Accessed June 30, 2026. https://www.nhsbsa.nhs.uk/statistical-collections/prescription-cost-analysis-england/prescription-cost-analysis-england-202324

[15] Office for National Statistics. Labour market overview, UK: employee earnings, December 2024. Accessed June 30, 2026. https://www.ons.gov.uk/employmentandlabourmarket/peopleinwork/employmentandemployeetypes/bulletins/uklabourmarket/december2024

[16] Herdman M, Gudex C, Lloyd A, . Development and preliminary testing of the new five-level version of EQ-5D (EQ-5D-5L). Qual Life Res. 2011;20(10):1727-1736. doi:10.1007/s11136-011-9903-x 21479777 PMC3220807

[17] Hernández Alava M, Pudney S, Wailoo A. Estimating the relationship between EQ-5D-5L and EQ-5D-3L: results from a UK population study. Pharmacoeconomics. 2023;41(2):199-207. doi:10.1007/s40273-022-01218-7 36449173 PMC9883358

[18] National Institute of Health Care and Excellence. Use of the EQ-5D-5L value set for England: NICE position statement. October 31, 2019. Accessed May 23, 2022. https://www.nice.org.uk/about/what-we-do/our-programmes/nice-guidance/technology-appraisal-guidance/eq-5d-5l

[19] Billingham L, Abrams KR, Jones DR. Methods for the analysis of quality-of-life and survival data in health technology assessment. Health Technol Assess. 1999;3(10):1-152. 10627631

[20] Devlin NJ, Shah KK, Feng Y, Mulhern B, van Hout B. Valuing health-related quality of life: an EQ-5D-5L value set for England. Health Econ. 2018;27(1):7-22. doi:10.1002/hec.3564 28833869 PMC6680214

[21] Ramsey S, Willke R, Briggs A, . Good research practices for cost-effectiveness analysis alongside clinical trials: the ISPOR RCT-CEA Task Force report. Value Health. 2005;8(5):521-533. doi:10.1111/j.1524-4733.2005.00045.x 16176491

[22] Barber JA, Thompson SG. Analysis of cost data in randomized trials: an application of the non-parametric bootstrap. Stat Med. 2000;19(23):3219-3236. doi:10.1002/1097-0258(20001215)19:23<3219::AID-SIM623>3.0.CO;2-P 11113956

[23] Lee Y, Nelder JA. Hierarchical generalised linear models: a synthesis of generalised linear models, random-effect models and structured dispersions. Biometrika. 2001;88(4):987-1006.

[24] White IR, Royston P, Wood AM. Multiple imputation using chained equations: issues and guidance for practice. Stat Med. 2011;30(4):377-399. doi:10.1002/sim.4067 21225900

[25] Faria R, Gomes M, Epstein D, White IR. A guide to handling missing data in cost-effectiveness analysis conducted within randomised controlled trials. Pharmacoeconomics. 2014;32(12):1157-1170. doi:10.1007/s40273-014-0193-3 25069632 PMC4244574

[26] Manca A, Hawkins N, Sculpher MJ. Estimating mean QALYs in trial-based cost-effectiveness analysis: the importance of controlling for baseline utility. Health Econ. 2005;14(5):487-496. doi:10.1002/hec.944 15497198

[27] Willan AR, Briggs AH, Hoch JS. Regression methods for covariate adjustment and subgroup analysis for non-censored cost-effectiveness data. Health Econ. 2004;13(5):461-475. doi:10.1002/hec.843 15127426

[28] Drummond MF, Sculpher MJ, Claxton K, Stoddart GL, Torrance GW. Methods for the Economic Evaluation of Health Care Programmes. Oxford University Press; 2015.

[29] Barton GR, Briggs AH, Fenwick EA. Optimal cost-effectiveness decisions: the role of the cost-effectiveness acceptability curve (CEAC), the cost-effectiveness acceptability frontier (CEAF), and the expected value of perfection information (EVPI). Value Health. 2008;11(5):886-897. doi:10.1111/j.1524-4733.2008.00358.x 18489513

[30] O’Neill B, Bradley JM, Connolly B, ; iRehab Trial Investigators. Remote multicomponent rehabilitation in intensive care unit survivors: a randomized clinical trial. JAMA. Published online May 18, 2026. doi:10.1001/jama.2026.7401 42150121 PMC13184783

[31] NHS England. Guidance for growing and developing the pulmonary rehabilitation multidisciplinary team. March 26, 2024. Accessed June 30, 2026. https://www.england.nhs.uk/long-read/pulmonary-rehabilitation-workforce/

[32] Berto PP, Teixeira C, Vianna MV, . The impact of mechanical ventilation on long-term survival influences definitions of persistent critical illness. Crit Care Sci. 2025;37:e20250388. doi:10.62675/2965-2774.20250388 40767694 PMC12266830

[33] Herridge MS, Azoulay É. Outcomes after critical illness. N Engl J Med. 2023;388(10):913-924. doi:10.1056/NEJMra2104669 36884324

[34] Gentile LF, Cuenca AG, Efron PA, . Persistent inflammation and immunosuppression: a common syndrome and new horizon for surgical intensive care. J Trauma Acute Care Surg. 2012;72(6):1491-1501. doi:10.1097/TA.0b013e318256e000 22695412 PMC3705923

[35] Yende S, Talisa VB, Mayes K, . Remote monitoring approaches to reduce readmissions after infection and sepsis: a randomized clinical trial. JAMA Netw Open. 2026;9(6):e2616641. doi:10.1001/jamanetworkopen.2026.16641 42275060 PMC13261490

[36] Wade DT. Health economic studies in clinical rehabilitation: a new collection of papers and a discussion of issues involved in research. Clin Rehabil. 2026;40(1):3-10. doi:10.1177/02692155251381743 41042939 PMC12722569

[37] Wade DT, Halligan PW. The biopsychosocial model of illness: a model whose time has come. Clin Rehabil. 2017;31(8):995-1004. doi:10.1177/0269215517709890 28730890

[38] Burge AT, Cox NS, Holland AE, . Telerehabilitation compared with center-based pulmonary rehabilitation for people with chronic respiratory disease: economic analysis of a randomized controlled clinical trial. Ann Am Thorac Soc. 2025;22(1):47-53. doi:10.1513/AnnalsATS.202405-549OC 39311774 PMC11708760

[39] Mosher CL, Nanna MG, Jawitz OK, . Cost-effectiveness of pulmonary rehabilitation among US adults with chronic obstructive pulmonary disease. JAMA Netw Open. 2022;5(6):e2218189. doi:10.1001/jamanetworkopen.2022.18189 35731514 PMC9218844

[40] Griffiths TL, Phillips CJ, Davies S, Burr ML, Campbell IA. Cost effectiveness of an outpatient multidisciplinary pulmonary rehabilitation programme. Thorax. 2001;56(10):779-784. doi:10.1136/thorax.56.10.779 11562517 PMC1745931

[41] Shields GE, Wells A, Doherty P, Heagerty A, Buck D, Davies LM. Cost-effectiveness of cardiac rehabilitation: a systematic review. Heart. 2018;104(17):1403-1410. doi:10.1136/heartjnl-2017-312809 29654096 PMC6109236

[42] Shields GE, Rowlandson A, Dalal G, . Cost-effectiveness of home-based cardiac rehabilitation: a systematic review. Heart. 2023;109(12):913-920. doi:10.1136/heartjnl-2021-320459 36849233

[43] Higgins AM, Lee YY, Bailey M, ; Treatment of Mechanically Ventilated Adults With Early Activity and Mobilization (TEAM) Study Investigators. The cost-effectiveness of early active mobilization during mechanical ventilation in the ICU: an economic evaluation alongside the Treatment of Mechanically Ventilated Adults With Early Activity and Mobilization (TEAM) Trial. Crit Care Med. 2025;53(9):e1725-e1735. doi:10.1097/CCM.0000000000006715 40439527 PMC12393053

[44] Tarride JE, Blackhouse G, Rochwerg B, ; Canadian Critical Care Trials Group. Cost-effectiveness of in-bed cycling and routine physiotherapy for patients receiving mechanical ventilation. JAMA Netw Open. 2025;8(9):e2529399. doi:10.1001/jamanetworkopen.2025.29399 40920382 PMC12418132

[45] Cuthbertson BH, Rattray J, Campbell MK, ; PRaCTICaL study group. The PRaCTICaL study of nurse led, intensive care follow-up programmes for improving long term outcomes from critical illness: a pragmatic randomised controlled trial. BMJ. 2009;339:b3723. doi:10.1136/bmj.b3723 19837741 PMC2763078

[46] Walsh TS, Salisbury LG, Merriweather JL, ; RECOVER Investigators. Increased hospital-based physical rehabilitation and information provision after intensive care unit discharge: the RECOVER randomized clinical trial. JAMA Intern Med. 2015;175(6):901-910. doi:10.1001/jamainternmed.2015.0822 25867659

[47] Bakhru R, Magee Bell C, Mazur J, Pfeifer C, Ford D. Implementation of a telehealth enabled post-ICU clinic. Am J Respir Crit Care Med. 2025;211(suppl 1):A1182. doi:10.1164/ajrccm.2025.211.Abstracts.A1182

[48] Sloan E, Parry SM, da Silva AA, . A systematic review of the development and implementability of complex interventions after hospitalization for survivors of intensive care. CHEST Crit Care. 2025;3(2):100142. doi:10.1016/j.chstcc.2025.100142

[49] Klaic M, Kapp S, Hudson P, . Implementability of healthcare interventions: an overview of reviews and development of a conceptual framework. Implement Sci. 2022;17(1):10. doi:10.1186/s13012-021-01171-7 35086538 PMC8793098

[50] Ramsey SD, Willke RJ, Glick H, . Cost-effectiveness analysis alongside clinical trials II—an ISPOR Good Research Practices Task Force report. Value Health. 2015;18(2):161-172. doi:10.1016/j.jval.2015.02.001 25773551

[51] Turner J, Knowles E, Simpson R, . Impact of NHS 111 Online on the NHS 111 telephone service and urgent care system: a mixed-methods study. Health Services and Delivery Research. 2021;9(21). doi:10.3310/hsdr09210 34780129

