# Supplementary material for: Remote Multicomponent Rehabilitation and Cost-Effectiveness in Survivors of Critical Illness
Source: JAMA Netw Open. 2026 Aug 10;9(8):e2628380. doi: 10.1001/jamanetworkopen.2026.28380 (PMC13458876; doi:10.1001/jamanetworkopen.2026.28380)
Supplement: Supplementary file 1 [file jamanetwopen-e2628380-s001.pdf]

Supplemental Online Content

Zanganeh M, O'Neill B, Bradley JM et al; iRehab Trial Investigators. Remote multicomponent rehabilitation and cost-effectiveness in survivors of critical illness. *JAMA Netw Open*. 2026;9(8):2628380. doi10.1001/jamanetworkopen.2026.28380

eAppendix 1. iRehab sites and acknowledged committees contributing to the iRehab study
eTable 1. Rehabilitation intervention costs per 231 patients
eTable 2. Unit cost of hospital inpatient admissions (patient and site reported)
eTable 3. Unit cost of hospital outpatient visits
eTable 4. Unit cost of Accident and Emergency (A&E) visits
eTable 5. Unit cost of special equipment/aids used
eTable 6. Unit cost of medication used (National Prescription Cost Analysis – 2023/24)
eTable 7. Unit cost of participant taken time off from work (Office for National Statistics [Labour market overview, UK: Employee earnings]) 2024
eTable 8. Unit cost of relatives/friends taken time off (Office for National Statistics [Labour market overview, UK: Employee earnings]) 2024
eAppendix 2. Justification regarding using missing at random (MAR) method for multiple imputation
eAppendix 3. A series of additional sensitivity analyses and sub-group analyses
eTable 9. Completeness of resource use and EQ-5D data by follow up visit
eTable 10. Health status; NHS PSS and societal costs for resource (Complete cases, unadjusted: baseline to 6 months) (Site-reported inpatient admission costs and Devlin et al algorithm for EQ-5D), (£, 2024)
eTable 11. Patient-reported inpatient admissions (complete cases, unadjusted)
eTable 12. Site-reported inpatient admissions (complete cases, unadjusted)
eTable 13. Health status (complete cases, adjusted)
eTable 14. Cost utility scenario analysis of different MNAR assumptions for missing cost and effectiveness quality-of-life data: Results at 6 months (£, 2024)
eFigure 1. Incremental net monetary benefit (NMB) across different WTP values for a QALY (NHS PSS perspective).
eFigure 2. Expected value of perfect information across different WTP values for a QALY (NHS PSS perspective).
eFigure 3. Cost-effectiveness acceptability curve showing the probability of rehabilitation intervention being cost-effective at different values of WTP for a QALY (Societal perspective).
eFigure 4. Cost-effectiveness acceptability curve showing the probability of rehabilitation intervention being cost-effective at different values of WTP for a QALY (ventilation duration <=7 days).
eFigure 5. Cost-effectiveness acceptability curve showing the probability of rehabilitation intervention being cost-effective at different values of WTP for a QALY (ventilation duration >7 days).
eReferences

This supplemental material has been provided by the authors to give readers additional information about their work.

© 2026 Zanganeh M et al. *JAMA Network Open*.

eAppendix 1. iRehab sites and acknowledged committees contributing to the iRehab study

The following 52 sites opened to recruitment for iRehab:

Aintree University Hospital, Liverpool, UK
Altnagelvin Hospital, Londonderry, UK
Birmingham Heartlands Hospital, Birmingham, UK
Craigavon Area Hospital, Craigavon, UK
Cumberland Infirmary, Carlisle, UK
Derriford Hospital, Plymouth, UK
Doncaster Royal Infirmary; Bassetlaw Hospital, Doncaster, UK
Freeman Hospital; Royal Victoria Infirmary, Newcastle-Upon-Tyne, UK
Glan Clwyd Hospital, Wales, UK
Glangwili General Hospital, Wales, UK
Good Hope Hospital, Birmingham, UK
Great Western Hospital, Swindon, UK
Hereford County Hospital, Hereford, UK
James Cook University Hospital, Middlesborough, UK
James Paget University Hospital, Norfolk, UK
King George Hospital, London, UK
Kingston Hospital, Kingston-Upon-Thames, UK
Manchester Royal Infirmary, Manchester, England
Musgrove Park Hospital, Taunton, UK
Newham University Hospital, London, UK
North Devon District Hospital, Barnstaple, UK
North Manchester General Hospital, Manchester, England
Northern General Hospital/Royal Hallamshire Hospital, Sheffield, UK
Pinderfields Hospital, Wakefield, UK
Princess Royal Hospital, Haywards Heath, UK
Queen Alexandra Hospital, Portsmouth, UK
Queen Elizabeth Hospital, London, UK
Queen's Hospital, Romford, UK
Rotherham Hospital, Rotherham, UK
Royal Berkshire Hospital, Reading, UK
Royal Blackburn Hospital, Blackburn, UK
Royal Free Hospital, London, UK
Royal Glamorgan Hospital / Prince Charles Hospital, Wales, UK
Royal Liverpool University Hospital, Liverpool, UK
Royal Oldham Hospital, Greater Manchester, UK
Royal Sussex County Hospital, Brighton, UK
Salford Royal Hospital, Manchester, UK
Southampton General Hospital, Southampton, UK
Stoke Mandeville Hospital, Aylesbury, UK
Sunderland Royal Hospital, Sunderland, UK
Ulster Hospital, Belfast, UK
University Hospitals Coventry and Warwickshire, Coventry, UK
Warrington Hospital, Warrington, UK
Watford General Hospital, London, UK
West Cumberland Hospital, Whitehaven, UK
West Suffolk Hospital, Bury Saint Edmunds, UK
Whipps Cross Hospital, London, UK

© 2026 Zanganeh M et al. *JAMA Network Open*.

Whiston Hospital, Prescot, UK
Whittington Hospital, London, UK
Wrexham Maelor Hospital, Wales, UK
Wythenshawe Hospital, Manchester, UK
Ysbyty Gwynedd Bangor, Wales, UK
iRehab was registered on the Associate PI Scheme, with six colleagues from sites completing this.

We thank the following committees contributing to the iRehab study:

Independent Data Monitoring and Ethics Committee (DMEC): Chair Dawn Teare, PhD (Biostatistics, Newcastle University), John Gormley, DPhil (Physiotherapy, Trinity College, Dublin), Catherine L Hough, MD (Pulmonary and Critical Care Medicine, Oregon Health and Science University).

Trial Steering Committee (TSC): Chair John Saxton, PhD (Clinical Exercise Physiology, University of Hull), Ben Creagh-Brown, PhD (Critical Care, University of Surrey and Royal Surrey NHS Foundation Trust), Sue Jowett, PhD (Heath Economics, University of Birmingham), Ioana Marian, MSc (Statistics, University of Oxford), Paul Chazot, PhD (Patient and Public Involvement and Biosciences, Durham University).

Patient Advisory Group (PAG): We would like to thank our Patient Advisory Group (PAG) members past and present for their inspiring contributions to this trial.

Trial Management Group (TMG): Brenda O'Neill PhD (Health Sciences, Ulster University), Danny McAuley MD (Critical Care Medicine, Queen's University Belfast), Julie Bruce PhD (Clinical Trials, University of Warwick), Gavin Perkins DSc (Critical Care Medicine, University of Warwick), Martin Underwood MD (Clinical Trials, University of Warwick), Paul Dark MD (Critical Care Medicine, University of Manchester), Nigel Hart MD (Biomedical Sciences, Queen's University Belfast), Ranjit Lall PhD (Statistics, University of Warwick), Chen Ji PhD (Statistics, University of Warwick), Mariam Ratna PhD (Statistics, University of Warwick), Mandana Zanganeh PhD (Health Economics, University of Warwick), Jason Madan PhD (Health Economics, University of Warwick), Katherine Jones PhD (Research Fellow, University of Warwick), Kerry Raynes MSc (Trial Manager, University of Warwick), Jill Costley BSc (Intervention Team, Ulster University), Annette Henderson BSc (Intervention Team, Ulster University), Darren Murphy MSc (Intervention Team, Ulster University), Rachel Clarke DClinPsy (Clinical Psychology, University Hospitals Plymouth NHS Trust), Cara Ghiglieri PhD (Process Evaluation Team, Queen's University, Belfast), Bronwen Connolly PhD (Process Evaluation Team, Queen's University Belfast), Judy Bradley PhD (Process Evaluation Team, Queen's University Belfast), Roger Kenyon HNC (Patient and Public Involvement).

Additional Acknowledgments (role): Rowena Williams MSc (TMG/Senior Project Manager, University of Warwick), Helen Bradley PhD (TMG/Senior Project Manager, University of Warwick), Emma Padfield (TMG/Senior Project Manager, University of Warwick), Radhika Kelkah (Document set-up/drafts, University of Warwick), Eva Schultz (telephone outcome assessment support, University of Warwick), Nicole De Valliere MSc (administration, telephone outcome assessment support, University of Warwick), Jessica Smith (TMG/Senior Project Manager, University of Warwick), Susie Hennings (TMG/Senior Project Manager, University of Warwick), Caroline Wilson PhD (Process evaluation, Queen's University Belfast), Kirsty Jerrard MSc (Intervention delivery, Ulster University), Niamh

© 2026 Zanganeh M et al. *JAMA Network Open*.

McCormac BSc (Intervention delivery, Ulster University), Kairenjit McCloy PhD (Intervention delivery, Ulster University), Michael Adams PhD (Intervention delivery, Ulster University), Alicia Clancy BSc (Intervention delivery, Ulster University), Louisa Edwards PhD (TMG/Trial Coordinator, University of Warwick), Raegan Barrows (University of Warwick), Sandra Baggott (TMG/Data Entry Clerk, University of Warwick), Sarah Lowe (TMG/Trial Manager, University of Warwick), Programming Team (Database development, reports, University of Warwick) and Quality Assurance Team (oversight, RAMP development, University of Warwick). All were compensated for their contributions.

© 2026 Zanganeh M et al. *JAMA Network Open*.

eTable 1. Rehabilitation intervention costs per 231 patients

	1:1 Appointments		Group/recorded exercise sessions		Online peer support group sessions (café)	
	**Average number of appointments attended**	**Average duration per appointment (minutes)**	**Average number of sessions attended**	**Average duration per session (minutes)**	**Average number of sessions attended**	**Average duration per session (minutes)**
	4.71	58.9	0.90	37.5	0.74	48.2
**Average number of appointments/sessions * average duration per appointment/session**	277.5		33.6		35.5	
**Cost per patient (£)**	£194.2[a]		£57.2[b]		£60.3[c]	
**Total cost of appointments/sessions per patient**	£312					
**Total cost of materials per patient**	£25[d]					
**Total cost from NHS PSS perspective per patient**	£337					
**Total societal cost per patient**	£83[e]					
**Total cost from societal perspective per patient**	£420					

[a] Cost (£) = 277.5*£0.7=£194.2. One staff with Physio background ran these appointments.
[b] Cost (£) = 33.6*£0.7+33.6*£1=£57.2. Two staff with physio background and non-physio background (Psychology or Sport and exercise science or Nursing) ran these sessions.
[c] Cost (£) = 35.5*£0.7+35.5*£1=£60.3. Two staff with physio background and non-physio background (Psychology or Sport and exercise science or Nursing) ran these sessions.

Unit cost of physio contact per hour and per minute respectively according to "Unit Costs of Health and Social Care 2024[1]" are £41 and £0.7. Unit cost of non-physio contacts per hour for Psychology or Nursing or Sport and exercise science respectively according to "Unit Costs of Health and Social Care 2024[1]" are £69, £58 and £54 (Averagely £60.3 per hour). Unit cost of non-physio contacts per minute for Psychology or Nursing or Sport and exercise science respectively according to "Unit Costs of Health and Social Care 2024[1]" are £1.1, £1 and £0.9 (Averagely £1 per minute).

[d] Cost (£) for one exercise manual, one recovery pack and two bookmarks for each patient in the intervention group was £3230 divided by the intervention participants (231) =£13.9. This was added by £3.5 cost of Royal mail postage and £7.5* of activity watches.

*Activity watches were sent to only 75 participants. For costing them across 231 participants, we multiplied 75 by £23 (cost of each of the activity watches) and divided by 231. Therefore, the cost of activity watches per patient in the intervention group was £7.5.

[e] Cost (£) = (277.5*£0.2)+(33.6*£0.2)+(35.5*£0.2)=£83.

Unit cost based on National Minimum Wage and National Living Wage rates 2024[2]: £11.44 per hour, £0.2 per minute.

© 2026 Zanganeh M et al. *JAMA Network Open*.

eTable 2. Unit cost of hospital inpatient admissions (patient and site reported)

Type of admission ward	Unit cost per episode (£), Planned			Source
	Regular day or night	Day case	Elective	
Cardiac	395	1933	7916	National Cost Collection Data Publication: National Schedule 2023-2024[3]
Digestive	326	885	6688	
Endocrine and metabolic	313	347	4709	
Haematology/Chemo/Cancer	415	507	5593	
Medical*	402	1031	6215	
Musculoskeletal/Orthopaedic	393	1700	8238	
Respiratory*	332	850	5242	
Surgical	402	1031	6215	
Urinary/Urology	298	1195	5716	
*For these categories considered an average of all planned admissions.				

Type of admission ward	Unit cost per episode (£), Unplanned		Source
	Non-Elective (short stay)	Non-Elective (long stay)	
Acute and general medical*	792	5134	National Cost Collection Data Publication: National Schedule 2023-2024[3]
Cardiac	691	4704	
Clinical decision*	792	5134	
Gastroenterology*	792	5134	
Hepatobiliary & Pancreatic	741	4740	
Infectious disease	597	4444	
Orthopaedic*	792	5134	
Respiratory	650	3963	
Surgical*	792	5134	
Trauma	1533	8945	
Urinary/Urology	696	4416	
*For these categories considered an average of all unplanned admissions.			

Type of admission ward	Unit cost per bed day (£), Adult Critical care	Source
Intensive Care Unit (ICU)	2276	National Cost Collection Data Publication: National Schedule 2023-2024[3]
Intensive Therapy Unit (ITU)		
High Dependency Unit		
**Type of admission ward**	**Unit cost up to 24-hour service (£), Emergency care**	**Source**
Accident & Emergency (A&E)	273	National Cost Collection Data Publication: National Schedule 2023-2024[3]

eTable 3. Unit cost of hospital outpatient visits

	Service	Reason	Unit cost (£) per attendance	Source
Outpatient visits	Cardiology	Vascular Ultrasound Scan*	216	National Cost Collection Data Publication : National Schedule 2023-2024[3]
		Echocardiogram (ECG)*	108	
		Vascular problem	186	
		Chest pain		
		Blood clot		
	Dermatology	Skin disease (Lupus)	173	
	Ear, nose and throat (ENT)	ENT check up	181	
		Hearing test		
	Endocrinology	Adrenal gland problem	291	
		Diabetes		
		Thyroid		
	Follow ups for a service	Follow up after A&E	296	
		Follow up after critical care (Intensive Care Unit)		
		Follow up visit (wound dressing, tube removal, post operation, after scan)		
	Gastroenterology	Upper Gastrointestinal endoscopy*	830	
		Colonoscopy*	807	
		Gastroenterology	223	
		Hepatology	234	
	Gynaecology	Breast clinic, Breast screening	222	
	Haematology	Haematology	297	
	Infectious disease	Sepsis	382	
		HIV check up		
		Infection		
	Neurology	Parkinson's	316	
		Epilepsy		
		Sleep apnoea		
		Stroke		
	Oncology	Radiotherapy	356	
		Chemotherapy		
	Orthopaedic	Bone density	172	
		Spinal, Shoulder, Leg, Feet		
	Ophthalmology	Eye check up	165	
	Psychology	Mental health	351	
		Depression		
	Radiology	X-ray: plain film (Chest, Knee, Kidney) *	43	
		CT scan (Lung, Brain, kidney, pelvis) *	127	
		Ultrasound scan (Thyroid, Kidney) *	230	
		MRI scan (Head)*	218	
	Rehabilitation	Rehab	362	
	Respiratory	Lung function problem	258	
		Chronic Obstructive Pulmonary Disease (COPD)		
		Pulmonary disease		
		Breathing problem		
		Pneumonia		
		Asthma		
	Rheumatology	Rheumatology	280	
	Speech/language	Speech/language	152	
	Surgery	Upper Gastrointestinal surgery	200	
		General surgery	179	
		Oral surgery	223	
	Urology	Renal	185	
		Urinary tract infection (UTI)		
	Unknown/other	Unknown	230	

For the ones not stated: average of consultant and non-consultant led, non-admitted f2f attendance. *Diagnostic imaging

© 2026 Zanganeh M et al. *JAMA Network Open*.

eTable 4. Unit cost of Accident and Emergency (A&E) visits

	Service	Reason	Unit cost (£) per attendance	Source
**Accident and Emergency (A&E) visits**	Cardiology	Low blood pressure	273	National Cost Collection Data Publication: National Schedule 2023-2024[3]
		High pulse rate		
		Chest pain		
		Echocardiogram (ECG)		
	Endocrinology	Pancreatitis		
		High blood sugar		
		Hypercalcemia		
	Gastroenterology	Vomiting		
		Diarrhoea		
		Severe constipation		
	Infection disease	Viral		
		Wound, throat		
		Sepsis		
		Infected lump		
	Neurology	Passed out		
	Ophthalmology	Orbital cellulitis		
		Eye haemorrhage		
		Detached retina		
	Orthopaedic	Wrist, Thumb, arm, hip		
	Psychiatry	Suicidal thought		
		Fatigue and depression		
	Radiology	MRI (spine)		
	Respiratory	Pneumonia		
		Breasting problem		
	Urology	Kidney problem		
		Urinary Tract Infection (UTI)		
		Stone in gallbladder		
		Blood loss in urine		

There is only one cost for all services/reasons.

© 2026 Zanganeh M et al. *JAMA Network Open*.

eTable 5. Unit cost of special equipment/aids used

	Type			Unit cost to NHS or social services (£)	Source
**Special equipment used**	Home adaptations (e.g. handrail or ramp)			Handrail or ramp: £55,<br>chair lift: £378	NHS Supply Chain (Aids for Daily Living) 2024[4]; Social care and support guide – NHS (Home adaptations)[5]
	Bathing aids			Toilet shower chair/commode;<br>Shower chair leaning stool/perching stool: £45,<br>Toilet frame: £50,<br>grab rail: £24	NHS Supply Chain (Aids for Daily Living) 2024[4]; Prestige, nursing and care[6]
	Stair lifts			£2,500	NHS Supply Chain (Aids for Daily Living) 2024[4]
	Wheelchair (manual/electric)			Manual: £153,<br>Electric: £343	Unit Costs of Health and Social Care 2024[1]
	Walking aids			Walking stick: £17,<br>Rollator/walking frame: £120,<br>Mobility scooter: £2750	NHS Supply Chain (Aids for Daily Living) 2024[4]; Social care and support guide – NHS (Walking aids, and mobility scooters)[7]
	Feeding aids			£80	NHS Supply Chain (Aids for Daily Living) 2024[4]
	Other	Cardio and respiratory aids	Blood pressure monitor	£30	NHS Supply Chain (Aids for Daily Living) 2024[4]
			Inhalers and spacer	£25	
			Nebuliser	£45	
			Oximeter	£25	
			Peak flow gadget	£15	
		Bed aids	Bed guard	£40	
			Hospital bed	£600	
			Inflatable mattress	£350	
		Others	PICO machine	£900	
			Treadmill and exercise bike	£200	
			Bio zoom machine	£100	
			Pov machine portable oxygen machine	£180	

© 2026 Zanganeh M et al. *JAMA Network Open*.

eTable 6. Unit cost of medication used (National Prescription Cost Analysis – 2023/24[8])

Medication name, dose, type	How long, frequency of use	Cost per item (£)	Quantity per item
**Alendronic acid 70mg tablets**	1 month, 1 per week	3.27	5
**Gabapentin 100mg capsules**	2 months, 4 per day;<br>4 months, 2 per day	26.53	280
**Gabapentin 300mg capsules**	2 months, 3 per day;<br>4 months, 1 per day	22+11=33	124
**Gabapentin 500mg/5ml oral liquid**	7 weeks, 3 per day	545.44+272.72=818.16	100
**Furosemide 40mg tablets**	8 weeks, 1 per day	2.17*2=4.34	28
	12 weeks, 1 per day	2.17*3=6.51	28
	4 months, 1 per day	2.17*4=8.68	28
**Levothyroxine sodium 25mcg tablets**	26 days, 1 per day	1.27*2=2.54	14
**Levothyroxine sodium 100mcg tablets**	2 months, 1 per day	1.95*2=3.3	30
**Bisoprolol 1.25mg tablets**	4 weeks, 1 per day	0.88	30
	2 months, 1 per day	0.88*2=1.76	30
	1 month, 3 per day	0.88*3=2.64	30
	4 months, 1 per day	0.88*4=3.52	30
	4 months, 2 per day	0.88*8=7.04	30
**Bisoprolol 2.5mg tablets**	8 weeks, 1 per day	5.18	62
	4 months, 1 per day	5.18*4=20.72	62
**Bisoprolol 5mg tablets**	8 weeks, 2 per day;<br>4 months, 1 per day	1.46*2=2.92	56
**Bisoprolol 10mg tablets**	2 months, 1 per day	0.83*2=1.66	30
	4 months, 2 per day	0.83*8=6.64	30
**Apixaban 2.5mg tablets**	1 month, 2 per day;<br>2 months, 1 per day	5.08*2=10.16	34
	2 months, 2 per day	5.08*4=20.32	34
	4 months, 2 per day	5.08*8=40.64	34
**Apixaban 5mg tablets**	1 month, 2 per day	31.28	60
	2 months, 2 per day;<br>4 months, 1 per day	31.28*2=62.56	60
	4 months, 2 per day	31.28*4=125.12	60
**Inhixa 120mg/0.8ml injection pre-filled syringes**	2 months, 1 per day	487.38	55
**Carbocisteine 250mg/5ml oral solution**	2 months, 3 per day;<br>4 months, 2 per day	9.75	300
**Carbocisteine 375mg capsules**	1 month, 2 per day	4.52	84
	4months, 2 per day	4.52*3=13.56	84
	4 months, 3 per day	4.52*4=18.08	84
**Carbocisteine 375mg capsules**	8 weeks, 3 per day	12.39	206
**Ferrous fumarate 210mg tablets**	8 weeks, 1 per day	0.82*2=1.64	28
	4 months, 1 per day	0.82*4=3.28	28
	4 months, 2 per day	0.82*8=6.56	28
**Ferrous fumarate 305mg capsules**	1 month, 1 per day	1.68	34
**Aspirin 75mg tablets**	2 months, 1 per day	2.88	84
	4 months, 1 per day	2.88*2=5.76	84
**Flucloxacillin 500mg capsules**	4 weeks, 2 per day	3.59 * 2=7.18	28
**Warfarin 3mg tablets**	2 months, 1 per day	1.74*2=3.48	30

© 2026 Zanganeh M et al. *JAMA Network Open*.

	4 months, 2 per day	1.74*8=13.92	30
**Warfarin 1.5mg/5m oral solution**	8 weeks, 1 per day	166.6	100
**Nefopam 30mg tablets**	2 months, 1 per day	3.57	90
	1 month, 6 per day	3.57*2=7.14	90
	4 months, 1 per day	3.57*3=10.71	90
**Acetylcysteine 600mg capsules**	1 month, 1 per day	24.95	30
**Thiamine 50mg tablets**	1 month, 2 per day;<br>2 months, 1 per day	1.23*2=2.46	40
	8 weeks, 4 per day;<br>4 months, 2 per day	1.23*6=7.38	40
**Thiamine 100mg tablets**	1 months, 3 per day	2.79+1.39=4.18	62
	2 months, 3 per day	2.79*3=8.37	62
	4 months, 3 per day	2.79*6=16.74	62
**Amoxicillin 250mg capsules**	1 week, 4 per day	1.98	28
**Metformin 500mg tablets**	4 weeks, 3 per day	3.75*7=26.25	14
	8 weeks, 1 per day	3.75*4=15	14
	3 months, 3 per day	3.75*6=22.5	14
**Vancomycin 125mg capsules**	10 days, 4 per day	126.16	40
**Insulin pen injection**	1 month, 1 per day	4.79	120
	2 months, 4 per day;<br>4 months, 2 per day	4.79*2=9.58	120
	4 months, 3 per day	4.79*3=14.37	120
**Prednisolone 5mg tablets**	3 weeks, 4 per day	3.34*2=6.68	42
	2 months, 1 per day	3.34*2=6.68	42
	4 months, 1 per day	3.34*3=10.02	42
**Omeprazole 20mg capsules**	8 weeks, 1 per day;<br>1 month, 2 per day	17.75*2=35.5	40
	2 months, 2 per day;<br>4 months, 1 per day	17.75*3=53.25	40
	4 months, 2 per day	17.75*6=106.5	40
**Omeprazole 40mg capsules**	8 weeks, 2 per day	6.85*4=27.4	30
	2 months, 1 per day	6.85*2=13.7	30
	4 months, 1 per day	6.85*4=27.4	30
	4 months, 2 per day	6.85*8=54.8	30
**Mycophenolate mofetil 1000mg oral suspension**	2 months, 4 per day	159.45	242
**Mycophenolate mofetil 500mg tablets**	2 months, 2 per day	197.42	120
	4 months, 2 per day	197.42*2=394.84	120
**Mirtazapine 15mg tablets**	1 month, 1 per day	1.77*2=3.54	30
	4 months, 1 per day	1.77*4=7.08	30
**Mirtazapine 30mg tablets**	2 months, 1 per day	1.11*2=2.22	30
**Edoxaban 30mg tablets**	2 months, 2 per day	122.5	70
**Edoxaban 60mg tablets**	2 months, 1 per day	53.75*2=107.5	30
**Aripiprazole 15mg tablets**	2 months, 1 per day	192.08	56
**Amitriptyline 10mg tablets**	8 weeks, 1 per day	1.34	60
	4 months, 1 per day	1.34*2=2.68	60
**Amitriptyline 25mg tablets**	2 months, 1 per day	1.89	64
**Amitriptyline 50mg tablets**	5 days, 1 per day	1	30

**Clopidogrel 75mg tablets**	2 months, 1 per day	1.35*2=2.70	30
	4 months, 1 per day	1.35*4=5.4	30
**Amlodipine 5mg tablets**	8 weeks, 1 per day	1.55	70
	2 months, 2 per day;<br>4 months, 1 per day	1.55*2=3.1	70
**Amlodipine 10mg tablets**	2 months, 1 per day	1.73	70
	4 months, 1 per day	1.73*2=3.46	70
**Voriconazole 200mg tablets**	4 weeks, 2 per day	1391.81	60
**Letrozole 2.5mg tablets**	1 months, 1 per day	93.45	30
	3 months, 1 per day	93.45*3=280.35	30
**Allopurinol 100mg tablets**	2 months, 2 per day;<br>4 months, 1 per day	1.7*2=3.4	60
**Allopurinol 300mg tablets**	1 month, 2 per day	1.24*2=2.48	30
**Loperamide 2mg capsules**	8 weeks, 4 per day	4.8*2=9.6	120
**Flexitol 10% Urea Cream**	2 days, 3 per day	7.63	260
**Codeine 15mg tablets**	2 weeks, 4 per day	1.83	60
**Codeine 30mg tablets**	8 weeks, 6 per day	3.47*4=13.88	90
**Spiriva Respimat inhaler**	2 months, 2 puffs per day;<br>4 months, 2 puffs per day	45.99	1
**Atorvastatin 10mg tablets**	1 week, 1 per day	1.37	30
**Atorvastatin 20mg tablets**	2 months, 1 per day	1.97*2=3.94	30
	3 months, 1 per day	1.97*3=5.91	30
	4 months, 1 per day	1.97*4=7.88	30
**Atorvastatin 40mg tablets**	2 months, 1 per day	1.94*2=3.88	30
	4 months, 1 per day	1.94*4=7.76	30
**Felodipine 5mg tablets**	1 month, 1 per day	4.38	30
**Sertraline 50mg tablets**	2 months, 1 per day	1.31+0.65=1.96	42
	2 months, 4 per day;<br>4 months, 2 per day	1.31*6=7.86	42
	4 months, 1 per day	1.31*3=3.93	42
**Lansoprazole 15mg tablets**	4 months, 1 per day	3.18*4=12.72	30
**Lansoprazole 30mg tablets**	2 months, 1 per day	5.68*2=11.36	30
	4 months, 1 per day	5.68*4=22.72	30
	4 months, 2 per day	5.68*8=45.44	30
**Creon 10000 capsules**	4 months, 6 per day	41.42*2=82.84	320
**Creon 25000 capsules**	2 months, 2 per day;<br>4 months, 1 per day	28.25*2=56.5	100
**Sacubitril 49mg/51 tablets**	3 weeks, 2 per day	99.14	60
	2 months, 2 per day	99.14*2=198.28	60
**Accrete D3 600mg/400IU tablets**	2 months, 2 per day	9.99*2=20.98	60
**Paracetamol 500mg tablets**	1 month, 3 per day	1.34	100
	2 months, 8 per day;<br>4 months, 4 per day	1.34*3=4.02	100
**Duloxetine 20mg tablets**	2 months, 2 per day	40.15*2=80.30	60
**Ramipril 1.25mg capsules**	2 months, 1 per day	1.30*2=2.60	30
	4 months, 1 per day	1.30*4=5.2	30
	4 months, 2 per day	1.30*8=10.4	30
**Ramipril 5mg capsules**	2 months, 2 per day;	3.13*2=6.26	64

© 2026 Zanganeh M et al. *JAMA Network Open*.

	4 months, 1 per day		
**Ramipril 10mg capsules**	2 months, 1 per day	1.28*2=2.56	30
	4 months, 1 per day	1.28*4=5.12	30
**Tacrolimus 5mg capsules**	4 weeks, 1 per day	205.74	50
**Co-codamol 30mg/500 capsules**	1 month, 4 per day	6	126
**Co-codamol 30mg/500 capsules**	2 months, 6 per day	6*3=18	126
**Nicotine patches**	2 weeks, 1 per day	9.6*2=19.2	7
**Levetiracetam 250mg sachets**	1 month, 2 per day	23	60
	4 months, 4 per day	23*4=92	60
**Levetiracetam 1000mg sachets**	2 months, 1 per day	76.27*2=152.52	60
	4 months, 2 per day	76.27*4=305.08	60
**Ursodeoxycholic 150mg capsules**	1 month, 2 per day	27.87	90
**Rifaximin 550mg tablets**	2 months, 2 per day	259.23*2=518.46	56
**Ticagrelor 90mg tablets**	2 months, 2 per day	59.98*2=119.96	60
	4 months, 2 per day	59.98*4=239.92	60
**Finasteride 5mg tablets**	1 month, 1 per day	3.03	30
	4 months, 1 per day	3.03*4=12.12	30
**Vitamin B12 100ml**	2 months, 2 per day	13.9	1
**Losartan 25mg tablets**	8 weeks, 1 per day	35.06	60
**Losartan 100mg tablets**	2 months, 1 per day	35.64	60
	4 months, 1 per day	35.64*2=71.28	60
**Fluorouracil 0.5% cutaneous solution**	1 week, 1 per day	39.05	25
**Fexofenadine 120mg tablets**	2 weeks, 1 per day	6.12	30
**Alogliptin 25mg tablets**	8 weeks, 1 per day	29.22	30
**Doxazosin 1mg tablets**	2 months, 1 per day	11.31*2=22.62	30
**Doxazosin 2mg tablets**	2 months, 1 per day	16.53*2=33.06	30
	2 months, 2 per day	16.53*4=66.12	32
**Hyoscine butylbromide 10mg tablets**	2 months, 8 per day	4.1*8=32.8	60
**Trimbow 87microg/dose / 5microg/dose / 9microg/dose inhale**	2 months, 2 puffs per day	44.5	1
**Avelle pump**	8 weeks, 1 per day	105.79	1
**Morphine 5mg orodispersible tablets sugar free**	2 months, 1 per day	3.85	60
	4 months, 1 per day	7.7	60
**Morphine 10mg orodispersible tablets sugar free**	2 months, 4 per day	5.27*4=21.08	62
**Amiodarone 200mg tablets**	2 months, 1 per day	3.65	64
**Gaviscon Double Action Liquid 10ml sachets**	2 months, 2 per day	21.07*2=42.14	60
	4 months, 2 per day	21.07*4=84.28	60
**Colchicine 500mcg tablets**	2 weeks, 1 per day	2.03	15
**Propranolol 10mg tablets**	2 months, 2 per day	9.24	168
	4 months, 2 per day	9.24*2=18.48	168
**Propranolol 40mg tablets**	2 months, 3 per day	2.76*3=8.24	56
	4 months, 3 per day	2.76*6=16.56	56
**Tamsulosin 400mcg capsules**	3 weeks, 1 per day	19.8	30
	2 months, 1 per day	19.8*2=39.6	30
**Naproxen 250mg tablets**	2 weeks, 2 per day	4.6	60

© 2026 Zanganeh M et al. *JAMA Network Open*.

**Evorel Sequi transdermal patches**	1 month, 1 every 3 days	32.12	20
**Fenofibrate 145mg tablets**	2 months, 1 per day	15.42	60
**Trelegy Ellipta 92mcg/55mcg/22mcg/dose inhale**	2 months, 1 puff per day	44.5	1
**Escitalopram 10mg tablets**	2 months, 1 per day	16.48*2=32.96	30
**Escitalopram 20mg tablets**	2 months, 1 per day	27.35*2=54.70	30
**Citalopram 40mg/ml oral drops sugar free**	4 months, daily	20.16	1
**Clonazepam 500mcg tablets**	2 months, 2 per day	17.81*2=35.62	60
**Co-trimoxazole 80mg/400mg tablets**	1 month, 1 per day	7.51	32
	4 weeks, 4 per day	7.51*4=30.04	32
	2 months, 3 per day	7.51*6=45.06	32
**Aciclovir 500mg tablets**	2 months, 3 per day	123.54*3=370.62	60
	2 moths, 2 per day	123.54*2=247.08	60
	4 months, 2 per day	123.54*4=494.16	60
**Doxazosin 1mg tablets**	1 month, 3 per day	11.31*3=33.93	30
**Famotidine 20mg tablets**	4 weeks, 1 per day	20.66	30
**Chloroquine phosphate 250mg tablets**	6 weeks, 1 per day	12.03*2=24.06	28
**Dapagliflozin 10mg tablets**	1 month, 2 per day;<br>2 months, 1 per day	39.61*2=79.22	30
	4 months, 1 per day	39.61*4=158.44	30
**Rivaroxaban 10mg tablets**	1 month, 1 per day	54	30
**Tinzaparin 8000units/0.4ml injection**	2 months, 2 per day	149.94*4=599.76	30
**Melatonin 2mg tablets**	2 months, 1 per day	14.17*2=28.34	30
	4 months, 1 per day	14.17*4=56.68	30
	4 months, 2 per day	14.17*8=113.36	30
**Spironolactone 12.5mg tablets**	1 week, 1 per day	8.4	14
	8 weeks, 2 per day	8.4*8=67.2	14
**Spironolactone 25mg tablets**	8 weeks, 1 per day	1.24*4=4.96	14
	4 months, 1 per day	1.24*8=9.92	14
**Spironolactone 100mg tablets**	2 months, 1 per day	24.9	70
**Pantoprazole 20mg gastro-resistant tablets**	8 weeks, 1 per day	5.48*2=10.96	32
**Lacosamide 100mg tablets**	1 month, 2 per day	89.59	58
	4 months, 1 per day	89.59*2=179.18	58
**Lacosamide 200mg tablets**	4 months, 2 per day	100.91*4=403.64	56
**Eplerenone 25mg tablets**	1 week, 1 per day	4.27	10
	2 months, 1 per day	8.34*2=16.68	32
	4 months, 1 per day	8.34*4=33.36	32
**Sodium chloride 600mg modified-release tablets**	2 weeks, 2 per day	6.25	70
**Solifenacin 10mg tablets**	2 months, 1 per day	1.82*2=3.64	30
**Ezetimibe 10mg tablets**	2 months, 1 per day	8.03*2=16.06	30
**Quetiapine 50mg modified-release tablets**	7 weeks, 1 per day	15.67*2=31.34	30
**Rimegepant 75mg oral lyophilisates sugar free**	8 weeks, 1 per day	390.99*2=781.98	30
**Folic acid 5mg tablets**	4 weeks, 1 per day;<br>2 months, 1 per day	0.93*2=1.86	32
	4 months, 1 per day	0.93*4=3.72	32
**Duloxetine 120mg gastro-resistant capsules**	2 months, 2 per day	24.19*4=96.76	34

© 2026 Zanganeh M et al. *JAMA Network Open*.

**Nitrofurantoin 50mg tablets**	2 days, 1 per day	3.31	7
**Cyanocobalamin 100mcg tablets**	1 month, 1 per day	2.08	32
**Digoxin 62.5mcg tablets**	8 weeks, 1 per day	3.92*2=7.84	30
**Digoxin 125mcg tablets**	10 weeks, 1 per day	3.81*3=11.43	30
	2 months, 1 per day	3.81*2=7.62	30
**Docusate 100mg capsules**	1 month, 2 per day	4.25	60
**Prochlorperazine 5mg tablets**	6 weeks, 3 per day	4.24*2=8.48	60
**Montelukast 10mg tablets**	2 months, 1 per day	3.7	60
	4 months, 1 per day	3.7*2=7.4	60
**Rosuvastatin 5mg tablets**	1 month, 1 per day	19.92	30
**Rosuvastatin 40mg tablets**	4 months, 1 per day	32.66*4=130.64	30
**Pregabalin 50mg capsules**	2 months, 2 per day	6.12*2=12.24	60
	4 months, 2 per day	6.12*4=24.48	60
**Pregabalin 100mg capsules**	5 weeks, 2 per day	6.31	75
**Candesartan 2mg tablets**	4 months, 1 per day	30.73*2=61.46	60
**Candesartan 4mg tablets**	2 months, 1 per day	21.02	60
**Fentanyl 50mcg/hour transdermal patches**	2 months, 1 every 3 days	119.25*2=238.5	10
**Entecavir 500mcg tablets**	2 months, every other day	363.26	30
**Salbutamol 100mcg/dose breath actuated inhaler**	2 months, 1 puff per day	10.04	1
**Mebeverine 135mg film-coated tablets**	1 week, 3 per day	44.28	50
**Lamotrigine 25mg tablets**	2 months, 2 per day	25.52*2=51.04	60
**Lamotrigine 100mg tablets**	4 months, 2 per day	69.04*4=276.16	56
**Generic Ensure Compact liquid**	1 month, 3 per day	38.71	1
**Perindopril erbumine 4mg tablets**	2 months, 1 per day	4.29	56
**Cyclizine 50mg tablets**	2 months, 3 per day	3.37*2=6.74	90
**Formoterol 12mcg/dose inhaler CFC free**	1 month, 1 puff per day	30.06	1
**Cetirizine 10mg tablets**	4 weeks, 1 per day	3	30
**Acetylcysteine 600mg tablets**	2 months, 1 per day	14.44*2=28.88	30
**Diazepam 2mg tablets**	4 weeks, 1 per day	0.79	30
**Hydroxyzine 25mg tablets**	2 months, 1 per day	3.74*2=7.48	30
	6 weeks, 3 per day	3.74*4=14.96	30
**Alfacalcidol 250nanogram capsules**	8 weeks, 1 per day	1.29*2=2.58	30
**Phenytoin sodium 100mg capsules**	2 months, 1 per day	3.3*2=6.6	30
**Lactulose 10g/15ml oral solution 15ml sachets sugar free**	1 month, 2 per day	7.5*2=15	30
**Phenoxymethylpenicillin 250mg tablets**	1 month, 2 per day	6.48	60
	4 months, 2 per day	6.48*4=25.92	60
**Sodium bicarbonate 500mg capsules**	6 weeks, 2 per day	1.32	84
**Generic Renapro powder 20g sachets**	1 month, 2 per day	139.2	60
**Colecalciferol 10,000unit capsules**	2 months, 1 per day	4.92*2=9.84	30
**Forceval capsules**	2 months, 3 per week	8.42	25
**Sodium valproate 500mg modified-release tablets**	1 month, 2 per day	12.22	60
**Olanzapine 5mg tablets**	1 month, 1 per day	0.93	30
**Optifibre powder**	2 weeks, 1 per day	9.88	28

**Gliclazide 40mg tablets**	2 weeks, 1 per day	0.24*2=0.48	7
	4 months, 1 per day	0.24*17=4.08	7
**Medroxyprogesterone 100mg tablets**	12 weeks, 2 per day	45.36*2=90.72	90
**Calcium carbonate 1.5g**	8 weeks, 1 per day	3.64*2=7.28	42
**Actonel 5mg tablets**	8 weeks, 1 per day	12.85*4=51.5	20
**Otrivine Adult 0.1% nasal drops**	5 days, 3 times per day	2.59	1
**Fluticasone propionate 50micrograms/dose nasal spray**	3 weeks, 2 times per day	5.53	1
**Trelegy Ellipta 92microg/55microg/22microg/dose dry inhale**	4 months, 1 per day	44.5	1
**Fostair 100micrograms/dose / 6micrograms/dose inhaler**	4 months, 4 times per day	32.59	1
**Ibuprofen 200mg capsules**	2 weeks, 4 per day	7.28	64
**Esomeprazole 20mg gastro-resistant tablets**	4 months, 1 per day	39.22*2=78.44	60
**Entresto 24mg tablets**	2 months, 1 per day	98.23	60
	4 months, 2 per day	98.23*4=392.92	60
**Fluoxetine 20mg tablets**	2 months, 1 per day	2.53	60
**Lisinopril 20mg tablets**	2 months, 1 per day	2.16	60
**Renavit tablets**	4 months, 1 per day	9.31	60
**Tramadol 100mg tablets**	3 months, 4 per day	2.78*12=33.36	30
**Oramorph 1mg/5ml syrup**	3 months, 4 times per day	5.41	1
**Trazodone 150mg tablets**	4 months, 1 per day	16.08*4=64.32	28
**Carvedilol 6.25mg tablets**	2 weeks, 1 per day	1.49	32
**Pizotifen 1.5mg tablets**	2 months, 1 per day	2.11*2=4.22	30
**Venlasov XL 75mg capsules**	1 week, 1 per day	2.98	28
**Evacal D3 1500mg/400unit chewable tablets**	4 months, 2 per day	2.96*4=11.84	60
**Ventolin 100micrograms/dose Evohaler**	4 months, 2 times per day	1.5	1
**Lantus 100units/ml injection 3ml pre-filled SoloStar pens**	27 days, 1 per day	41.75*4=167	6
**Timolol 10mg / Bendroflumethiazide 2.5mg tablets**	1 month, 1 per day	63.08	30
**Adoport 0.5mg tablet**	4 months, 4 per day	51.84*8=414.72	60
**Azathioprine 75mg tablets**	4 months, 2 per day	19.78*4=79.12	60
**Venlafaxine 150mg modified-release capsules**	3 months, 1 per day	17.4*3=52.2	28
**Valsartan 160mg capsules**	4 months, 2 per day	7.69*6=46.14	28
**Doxycycline 100mg capsules**	1 week, 2 per day	1.84	14

© 2026 Zanganeh M et al. *JAMA Network Open*.

eTable 7. Unit cost of participant taken time off from work (Office for National Statistics [Labour market overview, UK: Employee earnings]) 2024[9]

	Occupation	Salary gross (£)	Divide by 232 days (48 weeks * 5 days - 8 days): unit cost per day (£)
**Patients taken time off from work**	Administrator/coordinator	26,839	116
	Bank office clerk	28,259	122
	Builder	33,301	144
	Business Manager	56,484	243
	Butcher	28,081	121
	Care worker	25,570	110
	Catering Assistant	21,716	94
	Civil engineer	50,352	217
	Civil servant/public services associate professionals	39,537	170
	Cleaner	22,961	99
	Company secretary	33,131	143
	Computer analyst	35,146	151
	Counsellor/community worker	29,647	128
	Creative Director	45,970	198
	Customer Service Advisor	34,214	147
	Data Analyst	34,892	150
	Digital photographic restoration services (self-employed)	29,709	128
	Driver (bus)	34,548	149
	Driver (delivery/taxi)	26,995	116
	Driver (lift truck)	28,949	125
	Environment professional	37,219	160
	Export manager	35,665	154
	Family practitioner	48,288	208
	Finance Manager	75,093	324
	Financial administration	27,453	118
	Furniture Refurbishment	32,698	141
	Gardener	27,501	119
	Health and Safety Consultant/management (Self-employed)	42,461	183
	Health and social care assessor/worker	42,668	184
	Hotel receptionist	23,595	102
	IT service delivery/account manager	54,997	237
	Kitchen fitter	38,409	166
	Lawyer	51,624	223
	Lift engineer/Electrical	39,713	171
	Mechanic	35,540	153
	Medical Secretary	26,281	113
	Network operation technician	35,235	152
	Nurse	39,583	171
	Nursing assistant	28,041	121

© 2026 Zanganeh M et al. *JAMA Network Open*.

	Painter and decorator	32,036	138
	Patient Administrator	34,611	149
	Photographic Artist	38,169	165
	Plumber	38,099	164
	Postman/postal worker	33,583	145
	Psychotherapist	45,831	198
	Risk manager/watch manager	42,113	182
	Sales supervisor	26,603	115
	Sales/retail trainer	34,541	149
	Shop owner	33,674	145
	Stores Co-ordinator Network Rail	36,722	158
	Teacher	32,087	138
	Teacher Assistant	20,603	89
	Therapy assistant	31,804	137
	University Lecturer	48,521	209
	Web content technician	34,611	149
	Welder/metalworker	30,401	131
	Window fitter (Self-employed)	28,676	124

© 2026 Zanganeh M et al. *JAMA Network Open*.

eTable 8. Unit cost of relatives/friends taken time off (Office for National Statistics [Labour market overview, UK: Employee earnings]) 2024[9]

	Occupation	Salary gross (£)	Divide by 232 days (48 weeks * 5 days - 8 days): unit cost per day (£)	Unit cost (per hour) (£)
**Relatives taken time off from work**	Account manager	54,997	237	30
	Bank office clerk	28,259	122	15
	Builder	33,301	144	18
	Business manager	56,484	243	30
	Care worker	25,570	110	14
	Chemical analyst	32,779	141	18
	Civil engineer	50,352	217	27
	Civil servant/public services associate professionals	39,537	170	21
	Cleaner	22,961	99	12
	Community worker	29,647	128	16
	Company secretary	33,131	143	18
	Cook	22,282	96	12
	Customer Service Advisor	34,214	147	18
	Digital photographic restoration services (self-employed)	29,709	128	16
	Driver (bus)	34,548	149	19
	Driver (delivery/taxi)	26,995	116	15
	Financial administration	27,453	118	15
	Financial analyst	45,826	198	25
	Gardener	27,501	119	15
	Government administrative	30,295	131	16
	Health and social care assessor/worker	42,668	184	23
	Healthcare assistant	29,150	126	16
	Horticulturist	25,752	111	14
	Housing officer	32,934	142	18
	Human resource administrative	25,451	110	14
	IT service delivery/account manager	54,997	237	30
	Lab technician	27,058	117	15
	Lawyer	51,624	223	28
	Lift engineer	39,713	171	21
	Lift engineer/Electrical	39,713	171	21
	Mechanic	39,713	171	21
	Nurse	39,583	171	21
	Personal assistant	33,131	143	18
	Pharmacist	50,853	219	27
	Police officer	46,901	202	25
	Postman	33,583	145	18
	Sales/retail trainer	34,541	149	19
	Self-employed (carpenter/retail)	28,676	124	15

	Self-employed (management)	42,461	183	23
	Shop manager	33,674	145	18
	Teacher	32,087	138	17
	Teacher Assistant	20,603	89	11
	University Lecturer	48,521	209	26

© 2026 Zanganeh M et al. *JAMA Network Open*.

eAppendix 2. Justification regarding using missing at random (MAR) method for multiple imputation

Missing data are a common occurrence in studies collecting patient-level data. The choice of analytic method to be used for accounting for missing data depends on the extent of missing information and the likely underlying mechanism through which data were missing. Descriptive analyses of missing data were carried out to investigate the patterns of missing data (through graphs) and the likely mechanism of missingness. The appropriateness of using missing at random (MAR) was evaluated by investigating the missing data patterns and comparing attributes with and without missing utility and cost data at each follow-up time point. These comparisons did not raise any concerns about the appropriateness of the MAR assumption.

© 2026 Zanganeh M et al. *JAMA Network Open*.

eAppendix 3. A series of additional sensitivity analyses and sub-group analyses

The base-case analysis was complemented by a series of additional sensitivity analyses and sub-group analyses. We conducted sub-group analyses based on ventilation duration. Our planned sub-group analyses included trial randomisation strata with ventilation duration (≤7 vs >7 days) as a prespecified subgroup; this was based on the impact of prolonged mechanical ventilation on patient morbidity following critical illness [10-12]. We also carried out several sensitivity analyses to explore the impact of different sources of data, and specifications and assumptions in the cost-effectiveness analysis: (1) using societal perspective; (2) using site- reported inpatient admissions costs; (3) using Devlin et al. algorithm for EQ-5D[13]; (4) excluding intervention costs; (5) adjustment only for baseline utility; (6) using complete cases. In addition, to assess the robustness of cost-effectiveness results to departures from the MAR assumption, missing-not-at-random (MNAR) sensitivity analyses were implemented using pattern-mixture models. MNAR scenarios (best, worst) informed by discussion by the team. These scenarios were explored to evaluate the impact of plausible violations of MAR.

© 2026 Zanganeh M et al. *JAMA Network Open*.

eTable 9. Completeness of resource use and EQ-5D data by follow up visit

Parameter	Standard care	Rehabilitation intervention	Total
	n=198	n=231	n=429
	N (%)	N (%)	N (%)
**Health status (EQ-5D-5L)**			
Baseline	198 (100%)	231 (100%)	429 (100%)
8 weeks	167 (84.3%)	178 (77.1%)	345 (80.4%)
6 months	146 (73.7%)	168 (72.7%)	314 (73.2%)
Tota follow-up period	141 (71.2%)	160 (69.3%)	301 (70.2%)
**Data obtained through CRFs (NHS PSS perspective)**			
**Resource use (by time period)**			
Randomisation- 8 weeks	140 (70.7%)	144 (62.3%)	284 (66.2%)
8 weeks-6 months	125 (63.1%)	131 (56.7%)	256 (59.7%)
Total follow-up period	116 (58.6%)	115 (49.8%)	231 (53.8%)
**Resource use (by item)**			
**Inpatient**			
Inpatient admissions			
Randomisation- 8 weeks	149 (75.2%)	157 (68%)	306 (71.3%)
8 weeks-6 months	132 (66.7%)	141 (61%)	273 (63.6%)
Inpatient Nursing home admissions			
Randomisation- 8 weeks	146 (73.7%)	150 (64.9%)	296 (69%)
8 weeks-6 months	132 (66.7%)	140 (60.6%)	272 (63.4%)
Outpatient			
Outpatient visits			
Randomisation- 8 weeks	146 (73.7%)	156 (67.5%)	302 (70.4%)
8 weeks-6 months	131 (66.2%)	140 (60.6%)	271 (63.2%)
Accident & Emergency (A&E) visits			
Randomisation- 8 weeks	148 (74.7%)	152 (65.8%)	300 (69.9%)
8 weeks-6 months	132 (66.7%)	140 (60.6%)	272 (63.4%)
Outpatient Nursing home appointments			
Randomisation- 8 weeks	146 (73.7%)	150 (64.9%)	296 (69%)
8 weeks-6 months	132 (66.7%)	140 (60.6%)	272 (63.4%)
**Primary and community care**			
Randomisation- 8 weeks	146 (73.7%)	152 (65.8%)	298 (69.5%)
8 weeks-6 months	130 (65.6%)	140 (60.6%)	270 (62.9%)
**Social services used**			
Randomisation- 8 weeks	143 (72.2%)	151 (65.4%)	294 (68.5%)
8 weeks-6 months	129 (65.1%)	137 (59.3%)	266 (62%)
**Special equipment/aids used**			
Randomisation- 8 weeks	144 (72.7%)	151 (65.4%)	295 (68.8%)
8 weeks-6 months	128 (64.6%)	136 (58.9%)	264 (61.5%)
**Medication used**			

© 2026 Zanganeh M et al. *JAMA Network Open*.

Randomisation- 8 weeks	143 (72.2%)	146 (63.2%)	289 (67.4%)
8 weeks-6 months	128 (64.6%)	136 (58.9%)	264 (61.5%)
Data obtained through CRFs (Societal)			
Resource use (by time period)			
Randomisation- 8 weeks	136 (68.7%)	144 (62.3%)	280 (65.3%)
8 weeks-6 months	127 (64.1%)	131 (56.7%)	258 (60.1%)
Total follow-up period	114 (57.6%)	115 (49.8%)	229 (53.4%)
Resource use (by item)			
Private costs			
Special equipment/aids used			
Randomisation- 8 weeks	144 (72.7%)	151 (65.4%)	295 (68.8%)
8 weeks-6 months	128 (64.6%)	136 (58.9%)	264 (61.5%)
Private Health Services used			
Randomisation- 8 weeks	141 (71.2%)	147 (63.6%)	288 (67.1%)
8 weeks-6 months	128 (64.6%)	137 (59.3%)	265 (61.8%)
Travelling to health service providers			
Randomisation- 8 weeks	140 (70.7%)	146 (63.2%)	286 (66.6%)
8 weeks-6 months	128 (64.6%)	134 (58%)	262 (61.1%)
Work absence			
Participant taken time off from work			
Randomisation- 8 weeks	141 (71.2%)	147 (63.6%)	288 (67.1%)
8 weeks-6 months	129 (65.1%)	138 (59.7%)	267 (62.2%)
Relatives/friends taken time off			
Randomisation- 8 weeks	143 (72.2%)	145 (62.8%)	288 (67.1%)
8 weeks-6 months	129 (65.1%)	135 (58.4%)	264 (61.5%)
Extra Costs to Relatives/friends			
Randomisation- 8 weeks	141 (71.2%)	147 (63.6%)	288 (67.1%)
8 weeks-6 months	129 (65.1%)	135 (58.4%)	264 (61.5%)
Data obtained through CRFs (Societal perspective)			
Randomisation- 8 weeks	134 (67.7%)	140 (60.6%)	274 (63.87%)
8 weeks-6 months	124 (62.6%)	128 (55.4)	252 (58.7%)
Total follow-up period	111 (56.1%)	112 (48.5%)	223 (52%)

* Percentage calculated out of total randomised
CRF, case report form; NHS PSS, National Health Service Personal Social Service

© 2026 Zanganeh M et al. *JAMA Network Open*.

eTable 10. Health status; NHS PSS and societal costs for resource (Complete cases, unadjusted: baseline to 6 months) (Site-reported inpatient admission costs and Devlin et al algorithm for EQ-5D), (£, 2024)[a]

	n	Standard care Mean (SD)	n	Rehabilitation intervention Mean (SD)	Mean difference (95% CI)[b]	p-value [b]
**Health status (EQ-5D-5L)[c]**						
Baseline utilities	198	0.623 (0.242)	231	0.593 (0.290)	-0.030 (-0.080, 0.021)	.25
8 weeks utilities	167	0.674 (0.274)	178	0.691 (0.263)	0.017 (-0.040, 0.073)	.56
6 months utilities	148	0.673 (0.307)	169	0.712 (0.267)	0.039 (-0.027, 0.104)	.25
Area-under-the-curve (AUC), QALYs	143	0.341 (0.124)	161	0.344 (0.121)	0.003 (-0.024, 0.031)	.81
Costs for resource use categories (£)						
NHS PSS						
**Inpatient**						
Inpatient admissions [d]	198	£616.02 (2246.42)	231	£1342.43 (4400.44)	£726.41 (80.92, 1371.9)	**.03**
**All resource use costs (NHS PSS perspective)**						
Randomisation- 8 weeks	140	£966.12 (1261.49)	144	£1379.37 (1867.73)	£413.25 (49.01, 777.50)	**.03**
8 weeks-6 months	127	£843.23 (1080.75)	132	£1235.6 (1956.61)	£392.44 (18.51, 766.36)	**.04**
Total follow-up period	118	£1762.95 (1791.36)	115	£2588.22 (2876.86)	£825.2 (209.82, 1440.71)	**.009**
Total follow-up period + intervention cost (£337)	118	£1762.95 (1791.36)	115	£2925.22 (2876.86)	£1162.27 (547.51, 1777.02)	**P<.001**
**All resource use costs (Societal perspective)**						
Randomisation- 8 weeks	134	£4169.88 (4236.44)	140	£4132.68 (4010.71)	-£37.20 (-1000.30, 925.89)	.94
8 weeks-6 months	126	£4129.02 (7016.98)	129	£3655.65 (5438.47)	-£473.36 (-2040.31, 1093.58)	.55
Total follow-up period	113	£8601.25 (10346.94)	112	£7469.79 (8057.49)	-£1131.46 (-3598.04, 1335.12)	.37
Total follow-up period + intervention cost (£420)	113	£8601.25 (10346.94)	112	£7889.79 (8057.49)	-£711.46 (-3110.91, 1687.98)	.56

NHS PSS, National Health Service Personal Social Service; QALYs, quality adjusted life years
(n) in the table indicates the number of patients who provided data.
3 dead: [a] including three patients (one from rehabilitation and two from standard care) who died at follow up timepoint 6). The imputed and adjusted data of this Table includes two sensitivity analyses (see Table 6a).
[b] Mean difference (95% CI) and p-value calculated from bootstrapping using 2000 replications.
[c] Health status (EQ-5D-5L) calculated using Devlin et al algorithm[13].
[d] Site reported admissions: There were 26 admissions in the standard care group; and 58 admissions in the rehabilitation intervention group.

eTable 11. Patient-reported inpatient admissions (complete cases, unadjusted)

	n	Standard care Mean (SD)	n	Rehabilitation intervention Mean (SD)	n	Total Mean (SD)
**Inpatient**						
Inpatient admissions (baseline to 2 months)[a]	149	£574.52 (2756.68)	158	£685.52 (2571.19)	307	£631.64 (2659.04)
Inpatient admissions (2-6 months)[b*]	134	£437.11 (1510.74)	143	£1051.27 (3279.78)	277	£754.17 (2593.98)
Inpatient admissions (baseline to 6 months)[c*]	129	£900.95 (3173.17)	127	£1878.62 (5261.71)	256	£1385.97 (4355.93)

(n) in the table indicates the number of patients who provided data.

[a] There were **20** admissions for 11 patients in the standard care group (no ICU); and **18** admissions for 12 patients in the rehabilitation intervention group (including 2 ICU). Inpatient admissions for rehabilitation intervention group includes one serious adverse event (SAE).

[b] There were **15** admissions for 13 patients in the standard care group (no ICU); and **29** admissions for 18 patients in the rehabilitation intervention group (no ICU). Inpatient admissions for rehabilitation intervention group includes one serious adverse event (SAE).

[c] There were **35** admissions in the standard care group; and **47** admissions in the rehabilitation intervention group. Inpatient admissions for rehabilitation intervention group includes two serious adverse events (SAEs).

3 dead: * including three patients (one from rehabilitation and two from standard care) who died at FU timepoint 6).

© 2026 Zanganeh M et al. *JAMA Network Open*.

eTable 12. Site-reported inpatient admissions (complete cases, unadjusted)

	n	Standard care Mean (SD)	n	Rehabilitation intervention Mean (SD)	n	Total Mean (SD)
**Inpatient**						
Inpatient admissions (baseline to 2 months)[a]	198	£368.56 (1778.60)	231	£527.06 (2122.42)	429	£453.90 (1970.55)
Inpatient admissions (2-6 months)[b*]	198	£247.46 (1270.84)	231	£815.37 (3663.72)	429	£553.26 (2834.95)
Inpatient admissions (baseline to 6 months)[c*]	198	£616.02 (2246.42)	231	£1342.43 (4400.44)	429	£1007.17 (3586.09)

(n) in the table indicates the number of patients who provided data.

[a] There were **14** admissions for 13 patients in the standard care group (including 2 ICU and 1 HDU); and **23** admissions for 18 patients in the rehabilitation intervention group (including 2 ICU and 1 HDU).

[b] There were **12** admissions for 10 patients in the standard care group (including 1 ICU); and **35** admissions for 22 patients in the rehabilitation intervention group (including 1 ICU, 1 HDU).

[c] There were **26** admissions in the standard care group; and **58** admissions in the rehabilitation intervention group.

[c] **Standard care group:** Reasons for readmission (unplanned: 23: (medical ward, 8; respiratory ward, 3; cardiology ward, 0; surgical or orthopaedic ward, 3; other, 9) and (planned: 3). Timing during 6-week intervention period: 9; Timing after 6-week intervention period: 17.

[c] **Rehabilitation intervention group:** Reasons for readmission (unplanned: 48: (medical ward, 21; respiratory ward, 7; cardiology ward, 6; surgical or orthopaedic ward, 9; other, 5) and (planned: 10). Timing during 6-week intervention period: 21; Timing after 6-week intervention period: 37.

3 dead: * including three patients (one from rehabilitation and two from standard care) who died at FU timepoint 6).

© 2026 Zanganeh M et al. *JAMA Network Open*.

eTable 13. Health status (complete cases, adjusted)

	8 – week assessments				6 – month assessments			
Outcomes	Rehabilitation intervention	Standard care	Adjusted treatment estimate [a],[b] (95% CI)		Rehabilitation intervention	Standard care	Adjusted treatment estimate [a],[b] (95% CI)	
			Rehabilitation intervention v. Standard care	p-value			Rehabilitation intervention v. Standard care	p-value
***ITT analyses** [c]*								
**Health status**								
***Hernandez Alava[14]:* EQ-5D-5L index score[1], mean (SD), [N]**	0.60 (0.30) [178]	0.58 (0.31) [167]	0.05 (-0.004, 0.10)	0.073	0.63 (0.31) [169]	0.60 (0.33) [148]	0.07 (0.013, 0.12)[a]	.02
			0.05 (-0.004, 0.095)	0.069			0.06 (0.010, 0.117)[b]	.02
**Area-under-the-curve (AUC), QALYs**					0.300 (0.135) [161]	0.298 (0.139) [143]	0.022 (0.001, 0.041)[a]	.04
							0.021 (0.001, 0.040)[b]	.04
**Devlin et al[13].: EQ-5D-5L index score[1], mean (SD), [N]**	0.69 (0.26) [178]	0.67 (0.27) [167]	0.04 (-0.001, 0.09)	0.053	0.71 (0.27) [169]	0.67 (0.31) [148]	0.08 (0.03, 0.13)[a]	.003
			0.04 (0.001, 0.087)	0.043			0.07 (0.022, 0.12)[b]	.004
**Area-under-the-curve (AUC), QALYs**					0.344 (0.121) [161]	0.341 (0.124) [143]	0.023 (0.004, 0.041)[a]	.02
							0.022 (0.004, 0.039)[b]	.02
**EQ-5D-5L VAS[2], mean (SD), [N]**	67.29 (21.24) [178]	64.23 (21.25) [167]	2.59 (-1.37, 6.55)	0.199	68.86 (22.59) [169]	65.1 (23.89) [148]	4.01 (-0.71, 8.73)[a]	.09
			2.93 (-0.95, 6.81)	0.138			3.82 (-0.77, 8.41)[b]	.10

[a] Based on a generalized linear regression model adjusted for baseline utility score, stratification variables hospital sites and ventilation duration, and other covariates age, gender, ethnicity, and mobility; the therapist effect was negligible, and therefore no random effect was included to account for partial clustering.

[b] Based on a generalized linear regression model adjusted for baseline utility score.

[c] ITT=intention to treat, including three patients (one from rehabilitation and two from standard care) who died at 6-month FU timepoint.

1- EQ-5D-5L utility index scores range from less than 0 to 1 (0 indicates a health state equivalent to death and a negative value indicates worse than death). A higher score reflects better health utility.

2-EQ-5D-5L Overall health today scores range from 0 to 100. A higher score reflects better health.

eTable 14. Cost utility scenario analysis of different MNAR assumptions for missing cost and effectiveness quality-of-life data: Results at 6 months (£, 2024)

Scenario description	Incremental cost (£) (95%CI)	Incremental QALYs (95% CI)	ICER (£ per QALY)
**Best**			
-10% cost and +10% QoL in both arms	£1164 (523, 1805)	0.024 (0.007, 0.041)	49,135
**Worst**			
+10% cost and -10% QoL in both arms	£1336 (601, 2071)	0.022 (0.007, 0.038	59,920

ICER, Incremental cost-effectiveness ratio; MNAR, missing not at random; QALYs, quality adjusted life years
Based on a generalised linear regression model adjusted for baseline utility score, stratification variables hospital sites and ventilation duration, and other covariates age, gender, ethnicity, and mobility.
[a] Estimates derived from bootstrapping using 2000 replications (rehabilitation intervention compared to standard care)

© 2026 Zanganeh M et al. *JAMA Network Open*.

eFigure 1. Incremental net monetary benefit (NMB) across different WTP values for a QALY (NHS PSS perspective).

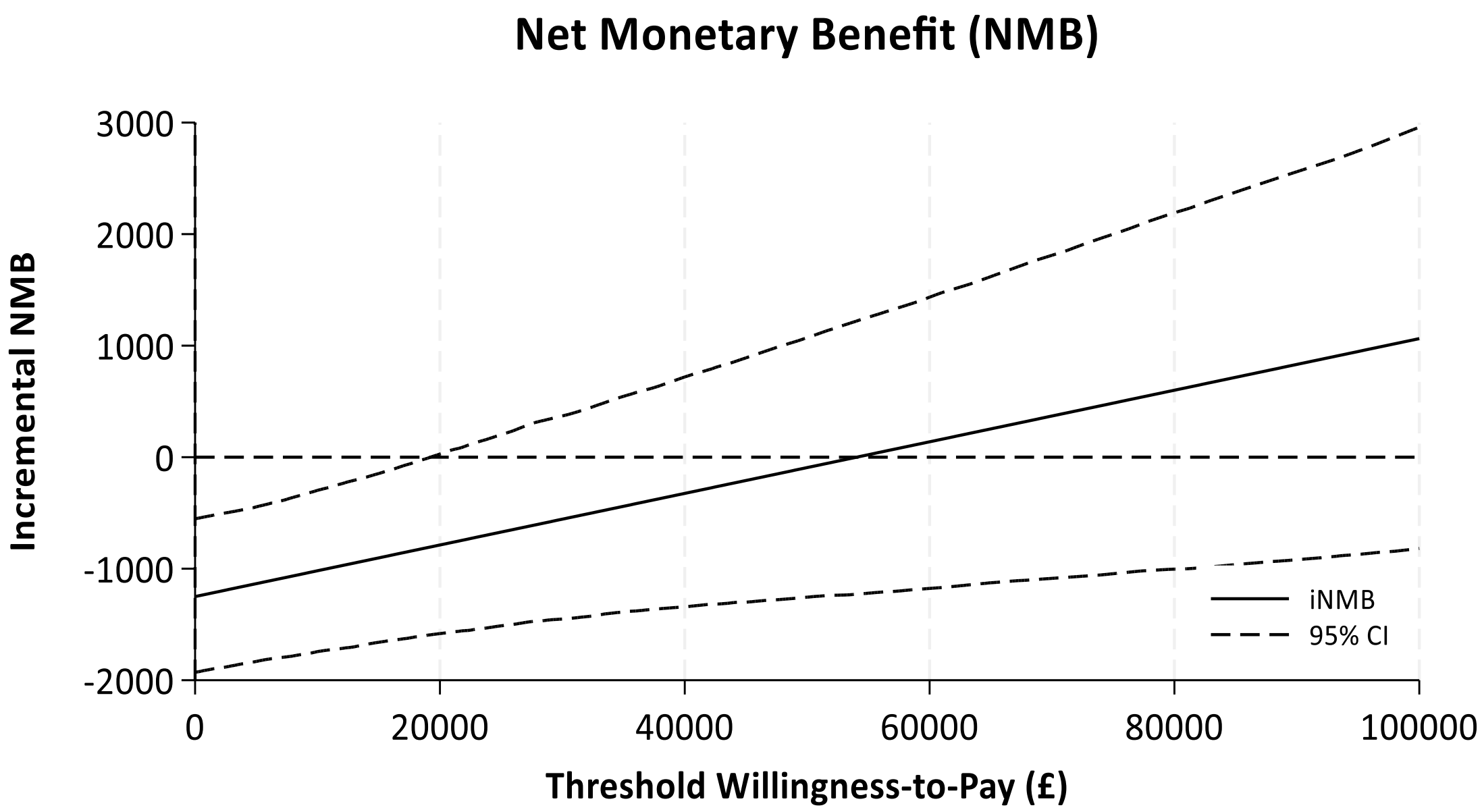

© 2026 Zanganeh M et al. *JAMA Network Open*.

eFigure 2. Expected value of perfect information across different WTP values for a QALY (NHS PSS perspective).

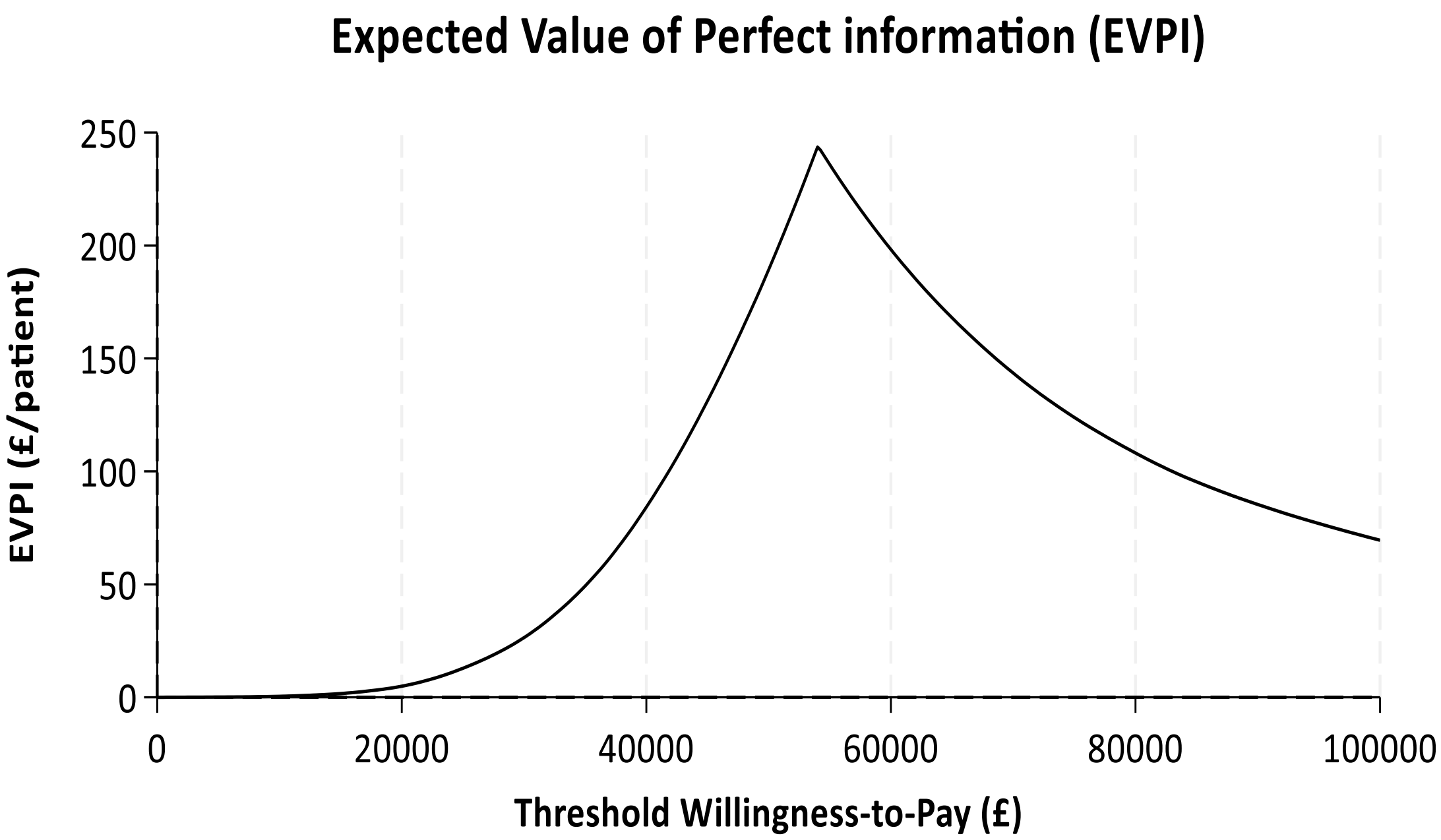

© 2026 Zanganeh M et al. *JAMA Network Open*.

eFigure 3. Cost-effectiveness acceptability curve showing the probability of rehabilitation intervention being cost-effective at different values of WTP for a QALY (Societal perspective).

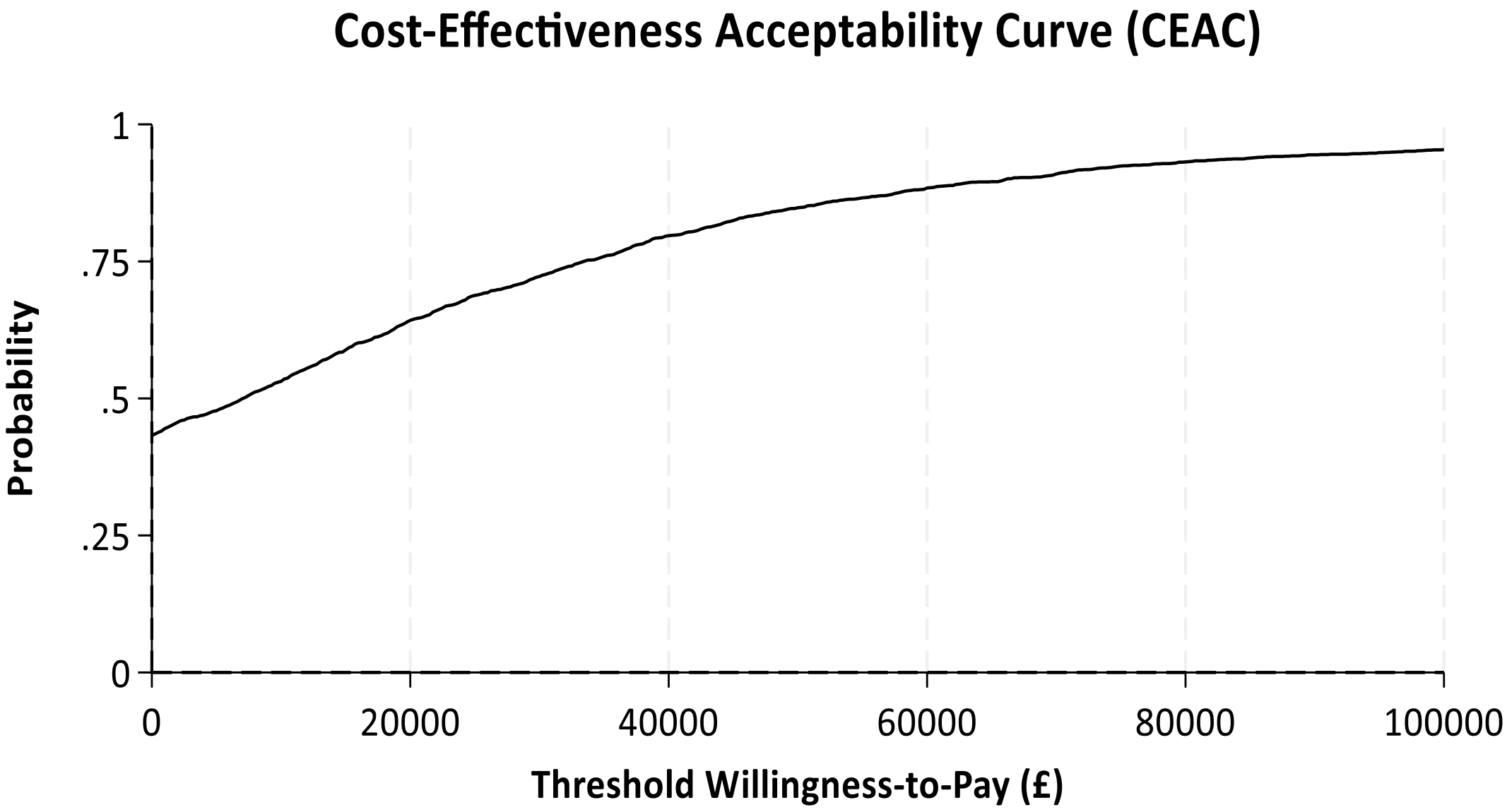

© 2026 Zanganeh M et al. *JAMA Network Open*.

eFigure 4. Cost-effectiveness acceptability curve showing the probability of rehabilitation intervention being cost-effective at different values of WTP for a QALY (ventilation duration <=7 days).

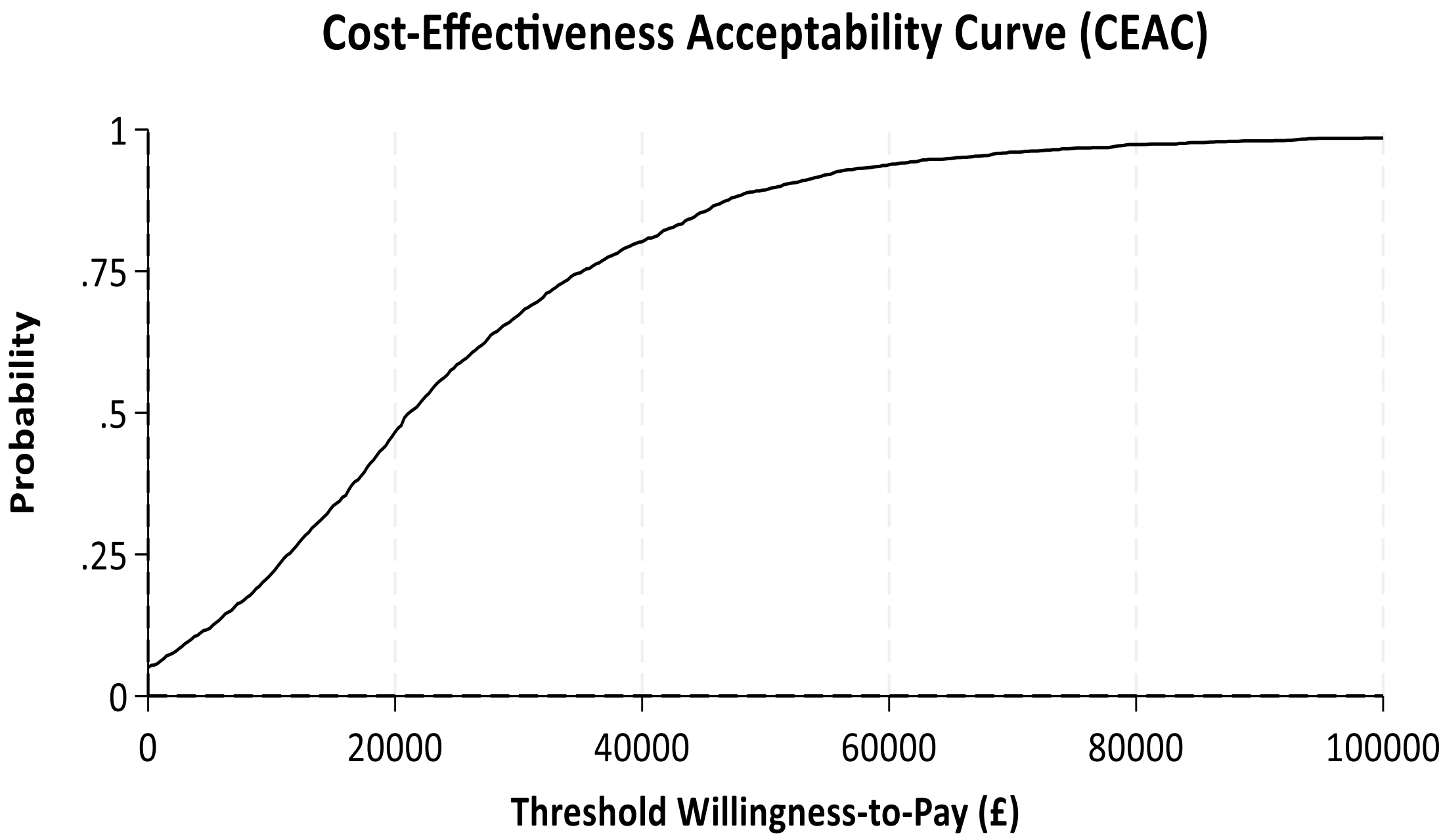

© 2026 Zanganeh M et al. *JAMA Network Open*.

eFigure 5. Cost-effectiveness acceptability curve showing the probability of rehabilitation intervention being cost-effective at different values of WTP for a QALY (ventilation duration >7 days).

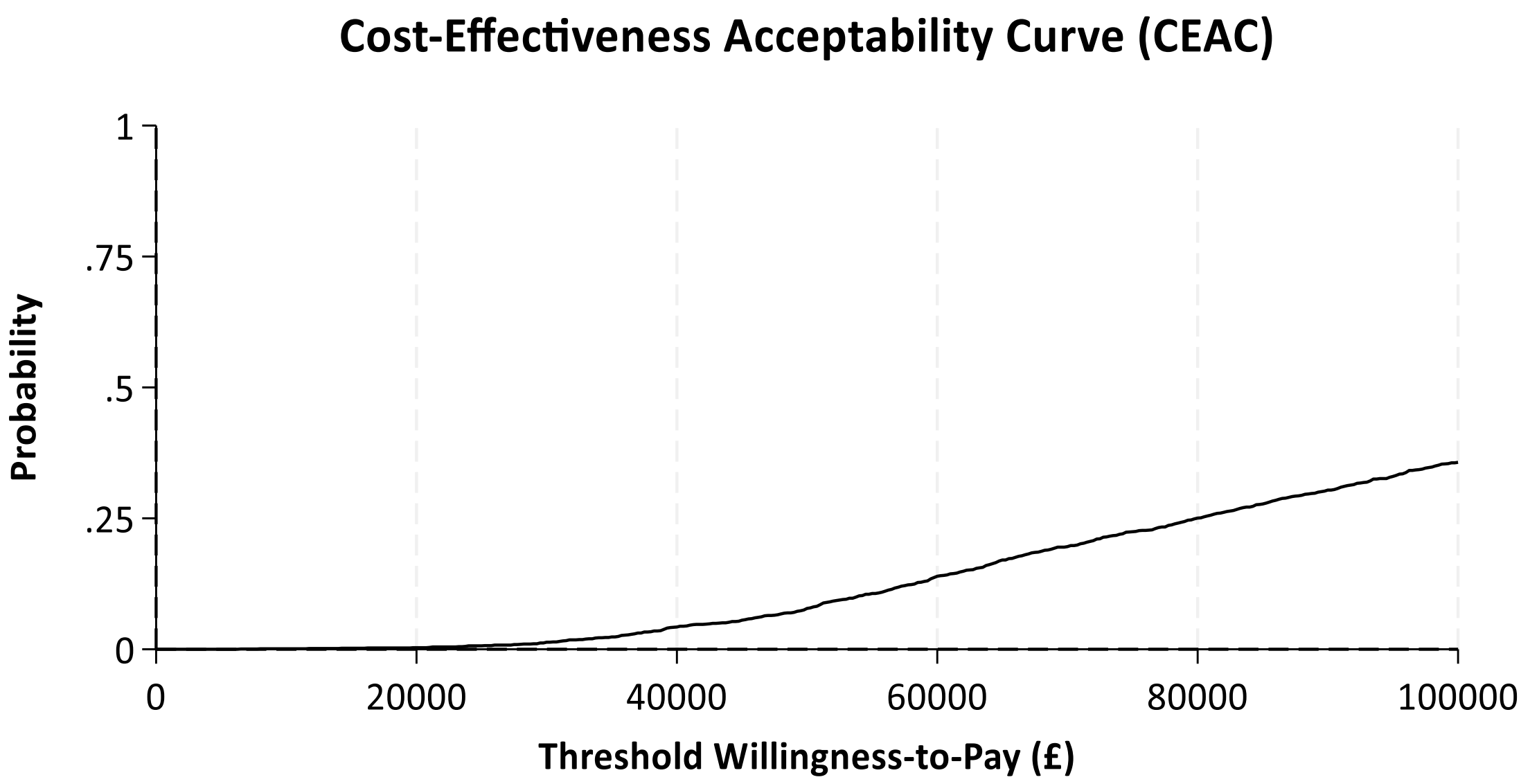

© 2026 Zanganeh M et al. *JAMA Network Open*.

eReferences

1. Jones KC, Weatherly H, Birch S, Castelli A, Chalkley M, Dargan A, et al. Unit Costs of Health and Social Care 2024 Manual [Available from: https://kar.kent.ac.uk/109563/.
2. National Minimum Wage and National Living Wage rates 2024 [Available from: https://www.gov.uk/national-minimum-wage-rates.
3. National Cost Collection Data Publication: National Schedule 2023-2024 [Available from: https://app.powerbi.com/view?r=eyJrIjoiNjI5YjA1OWMtOGNmOC00NGQzLWIxZDYtZDAyMDQ5YWVkZjU0IiwidCI6IjM3YzM1NGIyLTg1YjAtNDdmNS1iMjIyLTA3YjQ4ZDc3NGVlMyJ9.
4. NHS Supply Chain (Aids for Daily Living) 2024 [Available from: https://www.supplychain.nhs.uk/product-information/contract-launch-brief/walking-aids-and-simple-aids-for-daily-living/#delisted.
5. Social care and support guide – NHS (Home adaptations) [Available from: https://www.nhs.uk/social-care-and-support/care-services-equipment-and-care-homes/home-adaptations/.
6. Prestige, nursing and care [Available from: https://www.prestige-nursing.co.uk/health/the-top-8-bathing-aids-to-make-everyday-tasks-easier/.
7. Social care and support guide – NHS (Walking aids, and mobility scooters) [Available from: https://www.nhs.uk/social-care-and-support/care-services-equipment-and-care-homes/walking-aids-wheelchairs-and-mobility-scooters/.
8. National Prescription Cost Analysis – 2023/24 [Available from: https://www.nhsbsa.nhs.uk/statistical-collections/prescription-cost-analysis-england/prescription-cost-analysis-england-202324.
9. Office for National Statistics (Labour market overview, UK: Employee earnings) 2024 [Available from: https://www.ons.gov.uk/employmentandlabourmarket/peopleinwork/employmentandemployeetypes/bulletins/uklabourmarket/december2024.
10. O'Neill B, Bradley JM, Connolly B, Bruce J, Underwood M, Lall R, et al. Remote multicomponent rehabilitation compared to standard care for survivors of critical illness after hospital discharge (iRehab): a protocol for a randomised controlled assessor-blind clinical and cost-effectiveness trial. NIHR open research. 2025;5:29.
11. Herridge MS, Chu LM, Matte A, Tomlinson G, Chan L, Thomas C, et al. The RECOVER program: disability risk groups and 1-year outcome after 7 or more days of mechanical ventilation. American journal of respiratory and critical care medicine. 2016;194(7):831-44.
12. Berto PP, Teixeira C, Vianna MV, Rosa RG, Sganzerla D, Lisboa TC, et al. The impact of mechanical ventilation on long-term survival influences definitions of persistent critical illness. Critical Care Science. 2025;37:e20250388.
13. Devlin NJ, Shah KK, Feng Y, Mulhern B, van Hout B. Valuing health-related quality of life: An EQ-5D-5L value set for England. Health Econ. 2018;27(1):7-22.
14. Hernandez Alava M, Pudney S, Wailoo AJP. Estimating the relationship between EQ-5D-5L and EQ-5D-3L: results from a UK population study. 2023;41(2):199-207.

© 2026 Zanganeh M et al. *JAMA Network Open*.
