# Supplementary material for: Remote Multicomponent Rehabilitation and Cost-Effectiveness in Survivors of Critical Illness
Source: JAMA Netw Open. 2026 Aug 10;9(8):e2628380. doi: 10.1001/jamanetworkopen.2026.28380 (PMC13458876; doi:10.1001/jamanetworkopen.2026.28380)
Supplement: Supplementary file 2 [file jamanetwopen-e2628380-s002.pdf]

Supplemental Online Content: Nonauthor Collaborators
*First name, last name, and suffix (if applicable) are required and will appear in PubMed.

***Group Name(s): iRehab Trial Investigators**

***First Name and Middle Initial(s)**	***Last Name**	***Suffix (eg, Jr, III)**	Academic Degrees	Institution	Location (city, state/province, country)	Role or Contribution, eg, chair, principal investigator	Group (if more than 1 Group listed in the byline) and/or Subgroup (eg, Steering Committee)
Jane	Snell		MBChB	Aintree University Hospital	Liverpool, England	Principal investigator	
Sinead	O'Kane		PGCert	Altnagelvin Hospital	Londonderry, Northern Ireland	Principal investigator	
Jonathan	Weblin		MRes	Birmingham Heartlands Hospital	Birmingham, England	Principal investigator	
Denise	McFarland		BSc	Craigavon Area Hospital	Craigavon, Northern Ireland	Principal investigator	
Jane	Hedley		MSc	Cumberland Infirmary	Cumbria, England	Principal investigator	
Kate	Tantam		MRes	Derriford Hospital	Plymouth, England	Principal investigator	
Sarah	Varga		PhD	Doncaster Royal Infirmary	Doncaster, England	Principal investigator	
Catherine	Donnison		MSc	Freeman Hospital; Royal Victoria Infirmary	Newcastle Upon Tyne, England	Principal investigator	
Jennifer	Davies		BSc	Glan Clwyd Hospital	Denbighshire, Wales	Principal investigator	
Samantha	Coetzee		PGDip	Glangwili General Hospital	Carmarthen, Wales	Principal investigator	
Keeleigh	Champman		MSc	Great Western Hospital	Swindon, England	Principal investigator	
Amy	Arnold		BSc	Great Western Hospital	Swindon, England	Principal investigator	
Hannah	Hayter		BSc	Hereford County Hospital	Hereford, Herefordshire, England	Principal investigator	
Isabel	Gonzales		FRCA	James Cook University Hospital	Middlesbrough, England	Principal investigator	
Julie	North		BSc	James Paget University Hospital	Norfolk, England	Principal investigator	
Mandeep	Phull		FRCA	King George Hospital; Queen's Hospital	Ilford, England	Principal investigator	
Eriko	Morino		MBBCh	Kingston Hospital	Kingston upon Thames, England	Principal investigator	
Deborah	Paripoorani		MSc	Manchester Royal Infirmary	Manchester, England	Principal investigator	
Patricia	Doble		BSc	Musgrove Park Hospital	Somerset, England	Principal investigator	
Juan	Martin-Lazaro		PhD	Newham University Hospital	London, England	Principal investigator	
Charlotte	Tai		FRCP	Newham University Hospital	London, England	Principal investigator	
Ruth	Wood		BSc	North Devon District Hospital	Devon, England	Principal investigator	
Tracey	Hodgkiss		RGN	North Manchester General Hospital	Manchester, England	Principal investigator	
Kathryn	Birchall		RGN	Northern General Hospital	Sheffield, England	Principal investigator	
Eva	Hirst		PGCert	Pinderfields Hospital	Wakefield, England	Principal investigator	
Denise	Skinner		BSc	Princess Royal Hospital	Haywards Heath, England	Principal investigator	
Kayode	Adeniji		MSc	Queen Alexandra Hospital	Portsmouth, England	Principal investigator	
Estefania	Trues		MSc	Queen Elizabeth Hospital	London, England	Principal investigator	
Cheryl	Graham		BSc	Rotherham Hospital:	Rotherham, England	Principal investigator	
Francesca	Compton		BSc	Royal Berkshire Hospital	Reading, England	Principal investigator	
Nicholas	Truman		FFICM	Royal Blackburn Hospital	Blackburn, England	Principal investigator	
Lynn	McDonnell		BSc	Royal Free Hospital	London, England	Principal investigator	
Karthhika	Jeyakumar		BSc	Royal Free Hospital	London, England	Principal investigator	
Bethan	Gibson		MMBcH	Royal Glamorgan Hospital; Prince Charles Hospital	Llantrisant, Wales	Principal investigator	
Daniel	Law		BSc	Royal Glamorgan Hospital; Prince Charles Hospital	Llantrisant, Wales	Principal investigator	
Alicia	Waite		PhD	Royal Liverpool University Hospital	Liverpool, England	Principal investigator	

Supplemental Online Content: Nonauthor Collaborators
*First name, last name, and suffix (if applicable) are required and will appear in PubMed.

***First Name and Middle Initial(s)**	***Last Name**	***Suffix (eg, Jr, III)**	Academic Degrees	Institution	Location (city, state/province, country)	Role or Contribution, eg, chair, principal investigator	Group (if more than 1 Group listed in the byline) and/or Subgroup (eg, Steering Committee)
Rebecca	Chadwick		MSc	Royal Oldham Hospital	Greater Manchester, England	Principal investigator	
Daniel	Hansen		BA	Royal Sussex County Hospital	Brighton, England	Principal investigator	
Elizabeth	Howard		FICM	Salford Royal Hospital	Greater Manchester, England	Principal investigator	
Sarah	Howe		BSc	Salford Royal Hospital	Greater Manchester, England	Principal investigator	
Zoe	Van Willigen		MRes	Southampton General Hospital	Hampshire, England	Principal investigator	
Philip	Duggleby		FFICM	Stoke Mandeville Hospital	Buckinghamshire, England	Principal investigator	
Anthony	Rostron		PhD	Sunderland Royal Hospital	Sunderland, England	Principal investigator	
Samantha	Hagan		PGCert	Ulster Hospital	Belfast, Northern Ireland	Principal investigator	
David	McWilliams		PhD	University Hospitals Coventry and Warwickshire	Coventry, England	Principal investigator	
Joanna	Thomas		MRes	Warrington Hospital	Warrington, England	Principal investigator	
Ahmed	Abdelaty		FFICM	Watford General Hospital	Watford, England	Principal investigator	
Valerie	Page		FRCA	Watford General Hospital	Watford, England	Principal investigator	
Kirstin	Geer		MSc	West Cumberland Hospital	Cumbria, England	Principal investigator	
Suzahn	Wilson		MSc	West Suffolk Hospital	Bury St Edmunds, England	Principal investigator	
Martin	Udberg		MBChB	Whiston Hospital	Merseyside, England	Principal investigator	
Anezka	Pratley		DipHE	Whittington Hospital	London, England	Principal investigator	
Amy	Charnock		MSc	Wrexham Maelor Hospital	Wrexham, England	Principal investigator	
Luke	Ward		BNur	Wythenshawe Hospital	Manchester, England	Principal investigator	
Ellen	Knights		BSc	Ysbyty Gwynedd	Bangor, Wales	Principal investigator	
