# Supplementary material for: Remote Multicomponent Rehabilitation and Cost-Effectiveness in Survivors of Critical Illness
Source: JAMA Netw Open. 2026 Aug 10;9(8):e2628380. doi: 10.1001/jamanetworkopen.2026.28380 (PMC13458876; doi:10.1001/jamanetworkopen.2026.28380)
Supplement: Supplementary file 3 [file jamanetwopen-e2628380-s003.pdf]

Data Sharing Statement

Zanganeh. Remote Multicomponent Rehabilitation and Cost-Effectiveness in Survivors of Critical Illness. *JAMA Netw Open*. Published August 10, 2026.
doi:10.1001/jamanetworkopen.2026.28380

Data
Data available: Yes
Data types: Deidentified participant data, Data dictionary, Other (please specify)
Additional Information: Only anonymised data will be shared.
How to access data: wctudataaccess@warwick.ac.uk
When available: With publication

Supporting Documents
Document types: None

Additional Information
Who can access the data: Anyone can make an application and the WCTU Data Sharing Committee will, in conjunction with the CI and/or academic lead, consider the 5 safes around data sharing and will consider approvals by assessing the methodology and the experience of the applicant.
Types of analyses: For a specified purpose
Mechanisms of data availability: Electronic transfer of data
Any additional restrictions: The intended recipient would require appropriate ethical approval for the analysis
